# Host-*Mycobacterium avium* subsp. *paratuberculosis* interactome reveals a novel iron assimilation mechanism linked to nitric oxide stress during early infection

**DOI:** 10.1186/1471-2164-14-694

**Published:** 2013-10-10

**Authors:** Elise A Lamont, Wayne W Xu, Srinand Sreevatsan

**Affiliations:** 1 Department of Veterinary Population Medicine, College of Veterinary Medicine, University of Minnesota, 1971 Commonwealth Avenue, Saint Paul, MN 55108, USA; 2 Department of Veterinary Biomedical Sciences, University of Minnesota, Saint Paul, MN 55108, USA; 3 Minnesota Supercomputing Institute, University of Minnesota, Saint Paul, MN 55108, USA

**Keywords:** *Mycobacterium avium* subsp. *paratuberculosis*, Johne’s disease, RNA-seq, Mycobacteria, Bovine, Macrophage, Epithelium, Co-culture, Interactome, Iron

## Abstract

**Background:**

The initial interaction between host cell and pathogen sets the stage for the ensuing infection and ultimately determine the course of disease. However, there is limited knowledge of the transcripts utilized by host and pathogen and how they may impact one another during this critical step. The purpose of this study was to create a host-*Mycobacterium avium* subsp. *paratuberculosis* (MAP) interactome for early infection in an epithelium-macrophage co-culture system using RNA-seq.

**Results:**

Establishment of the host-MAP interactome revealed a novel iron assimilation system for carboxymycobactin. Iron assimilation is linked to nitric oxide synthase-2 production by the host and subsequent nitric oxide buildup. Iron limitation as well as nitric oxide is a prompt for MAP to enter into an iron sequestration program. This new iron sequestration program provides an explanation for mycobactin independence in some MAP strains grown in vitro as well as during infection within the host cell. Utilization of such a pathway is likely to aid MAP establishment and long-term survival within the host.

**Conclusions:**

The host-MAP interactome identified a number of metabolic, DNA repair and virulence genes worthy for consideration as novel drug targets as well as future pathogenesis studies. Reported interactome data may also be utilized to conduct focused, hypothesis-driven research. Co-culture of uninfected bovine epithelial cells (MAC-T) and primary bovine macrophages creates a tolerant genotype as demonstrated by downregulation of inflammatory pathways. This co-culture system may serve as a model to investigate other bovine enteric pathogens.

## Background

Molecular cross talk has recently been referred to as a dance of seduction that encompasses various tissues, cells and effector molecules [1,2]. This dance particularly holds true for the host-pathogen dynamic at the intestinal epithelium interface [3]. One common stratagem that intestinal pathogens utilize is to take advantage of or misdirect normal cell-to-cell cross talk that occurs between the intestinal epithelium and subepithelial dome (SED) macrophages [4-11].

Few studies focus on epithelium-*Mycobacterium avium* subsp. *paratuberculosis* (MAP) interactions and go beyond MAP translocation through M cells due to the lack of *in vitro* and animal models that recapitulate pathogenesis [5,6,12-17]. Therefore, the majority of host-MAP studies center on the macrophage in part due to the intracellular lifestyle of pathogenic mycobacteria and the designation of enterocytes as bystander cells (until recently) [16,18-22]. However, we and others have shown that the epithelium plays an active role during early infection with MAP and that epithelium processing of MAP may greatly contribute to the course of infection. Epithelium processing and interaction with bovine mammary epithelial cells (MAC-T), a surrogate for the intestinal epithelium, results in enhanced phagocytosis during secondary infection [23]. Changes to invasion phenotype due to epithelial processing are also seen with *M. smegmatis*, a saprophytic mycobacteria that is nonpathogenic [24]. *M. smegmatis* exposed to A549 epithelial cells had a significant increase in intracellular growth during secondary infection in THP-1 macrophages [24]. Epithelium processing of MAP may also impact which repertoire of pathogen genes are used during infection to promote its survival in its target cell, the macrophage. For example, MAP invasion into Madin-Darby Bovine Kidney (MDBK) cells, another surrogate cell type for the intestinal epithelium, upregulated an oxidoreductase (MAP3464) to regulate the Cdc42 pathway [25]. The Cdc42 pathway is also regulated by other pathogens to form filopodia and cytoskeleton rearrangement [26,27]. We have shown that MAP transcriptional profiles isolated and enriched from the ileum (IL) and mesenteric lymph nodes (MLN) from naturally infected cattle are significantly divergent from straight macrophage infection [28]. A number of reasons for this exists which includes 1) epithelial processing of MAP and 2) cross talk between the epithelium and macrophage. For instance, cross talk between the epithelium and macrophage results in the downregulation of pathogen recognition receptors (eg. Toll-like receptors 2 and 4), which creates an inflammation anergic state in intestinal macrophages and may impact which genes are needed by MAP to survive [29-32]. More recently, we have elucidated a mechanism for MAP orchestrated macrophage transepithelial migration that is reliant on phagosome maturation concomitant with IL-1β production at the epithelial interface during early infection [33]. Taken together these data suggest that MAP’s first interaction within the host at the intestinal epithelium interface is a dynamic process that can be harnessed by the pathogen to achieve survival and dissemination within the macrophage. This interaction is unnoticed in *in vitro* macrophage models alone and it is likely that the MAP encountered by SED and lamina propria macrophages shows an entirely different transcriptional and proteomic profiles. Consequently, reported studies utilizing macrophage infection models are suspected to underestimate both host and MAP responses.

In order to bridge the knowledge gap between pathogen processing by various cell types and multiple layers of cross talk, a host-pathogen interactome must be established [34]. Based on our previous study using the epithelium/macrophage co-culture system during MAP infection, we expect that elucidation of the early molecular events resultant from multiple layers of cross talk is critical to understanding pathogen establishment and survival within the host. Host pathways involved during infection are expected to be influenced by cell-to-cell crosstalk and release of extrinsic factors. MAP processed by the epithelium prior to macrophage infection may utilize a different set of genes in comparison to macrophage infection a priori. This is the first study to show that pathways involved during early stages of MAP infection are influenced by pathogen processing by the epithelium and cell-to-cell cross talk. These results show an active role of the epithelium in establishment of MAP infection, which augments our knowledge of MAP pathogenesis as well as sheds light on pathways for disruption in novel vaccine designs.

## Results and discussion

**Figure 1:**
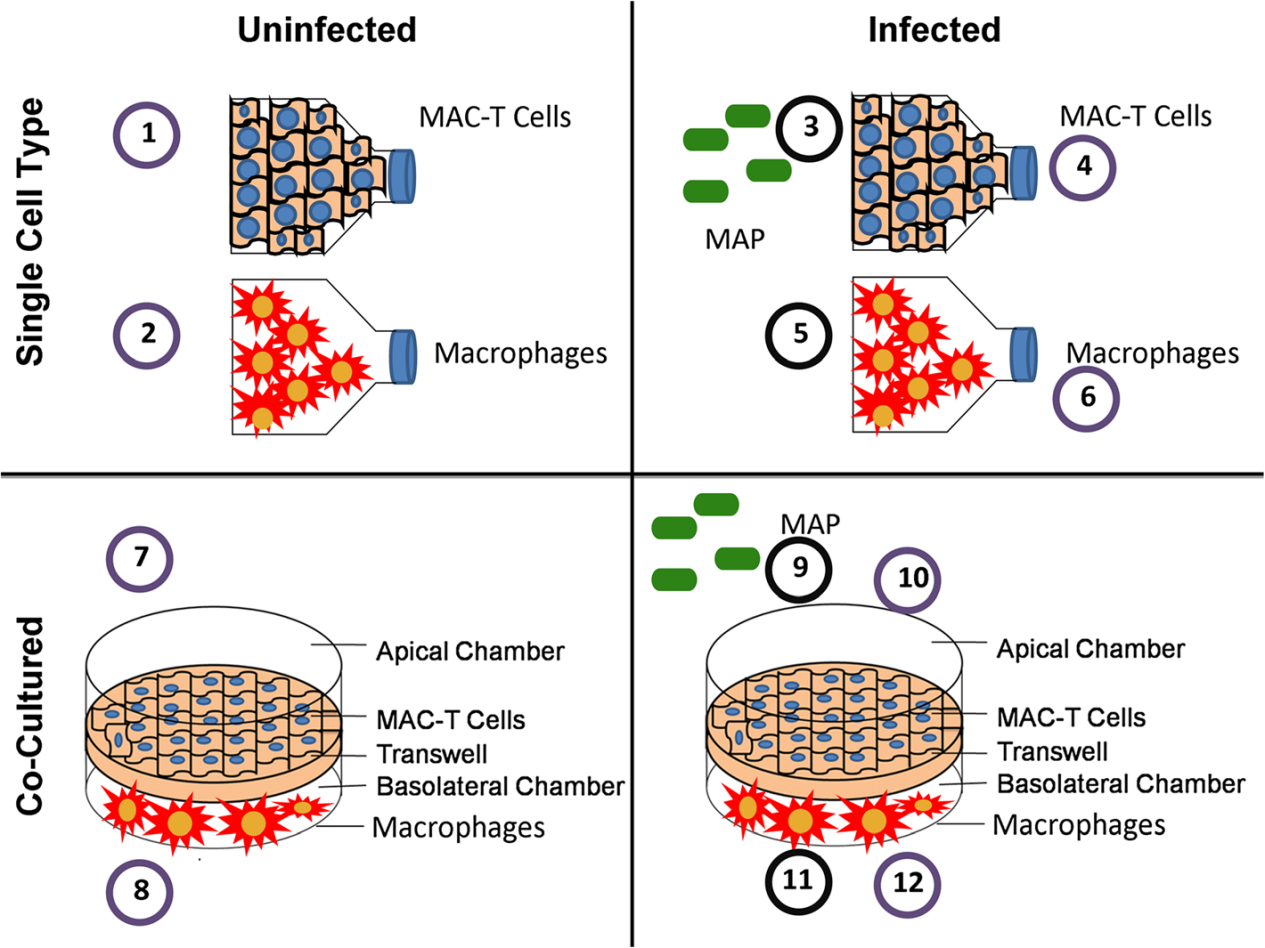
**Experimental setup.** MAC-T cells (1) and macrophages (2) were cultured separately or co-cultured (7 and 8, respectively) at a density of ~ 2.0 × 10^4^ cells/mL under uninfected (1, 2, 7 and 8) or MAP infected conditions (4, 6, 10 and 11). RNA was extracted from host cells (1, 2, 4, 6, 10 and 12) and pathogen located inside MAC-T cells (3 and 9) and macrophages (5 and 11) at 30 min post-infection. A total of 12 conditions (samples) were submitted for RNA-seq analysis.

Infection under co-culture of different types of cells will reveal both the cell cross-talk and the host and pathogen interaction. In this study, macrophages and MAC-T cells were cultured alone or together in a transwell system and subjected to MAP infection at 30 min. post-infection, which allowed for epithelium processing of MAP as well as multiple layers of cross-talk (Figure 1). RNA-seq was conducted on MAC-T, macrophages alone, or co-culture, with or without MAP infection. A total of 86,622,230 pair 76-base sequence reads were generated from 8 host samples and passed the quality check. More than 80% of reads were mapped on bovine reference genome. 74,019,002 pair 76-base sequence reads were generated from 4 infected and bacterial-enriched samples and approximately 15% of these reads were mapped on MAP reference genome.

### Extrinsic factors released by macrophages inhibit cellular growth to prevent cell death in MAC-T cells under co-culture conditions

**Figure 2:**
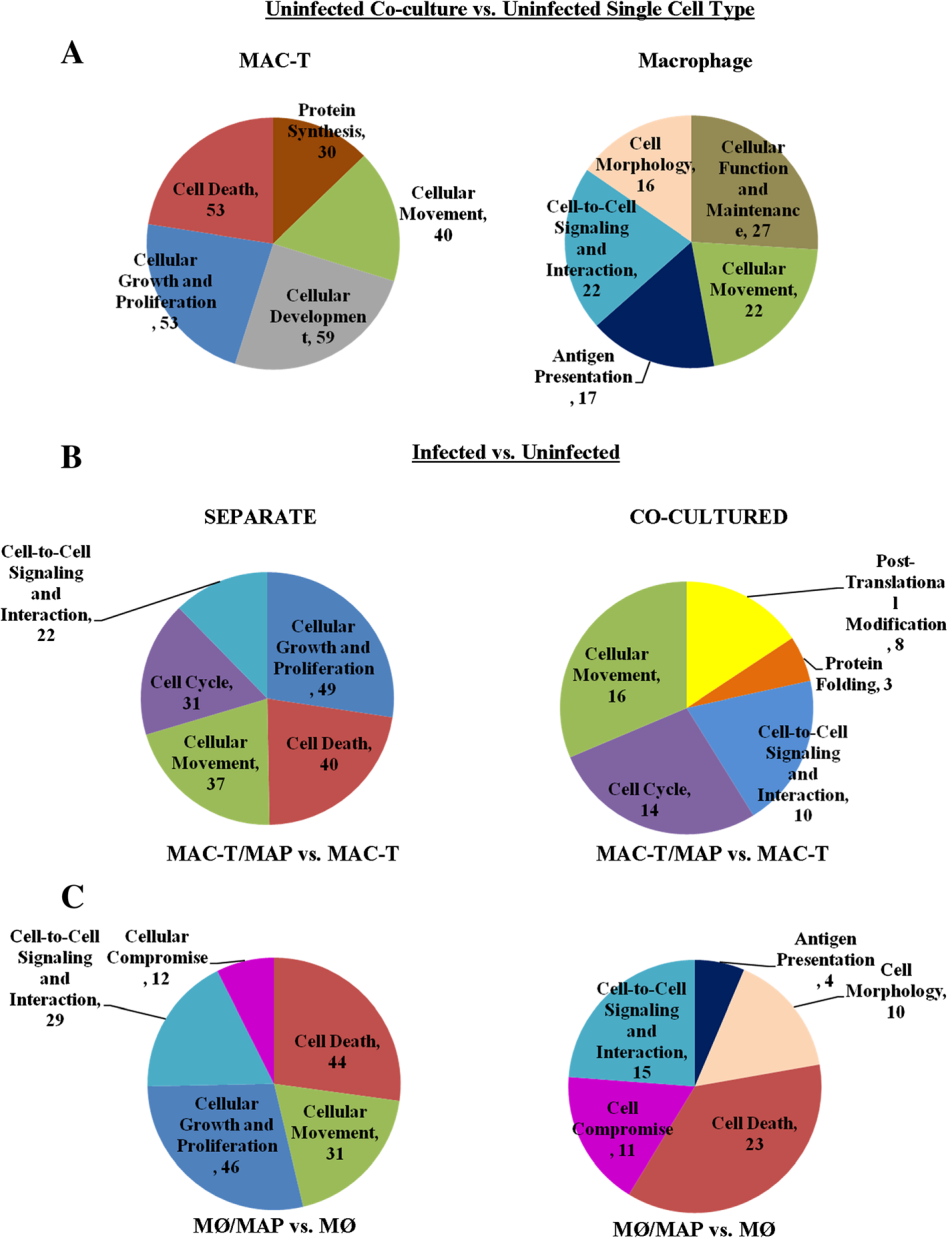
**Cellular and molecular functions identified by differential gene expression.** Identified host transcripts (P < 0.05) were submitted to IPA for molecular/cellular function analyses. Genes were mapped against human, mouse and rat genomes for homology. Molecular/cellular functions and number of genes for **A)** uninfected co-cultured cells vs. uninfected single cell types, **B)** MAC-T cells cultured alone (separate) or co-cultured infected with MAP vs. corresponding uninfected control, and **C)** macrophages cultured alone (separate) or co-cultured infected with MAP vs. corresponding uninfected control.

**Figure 3:**
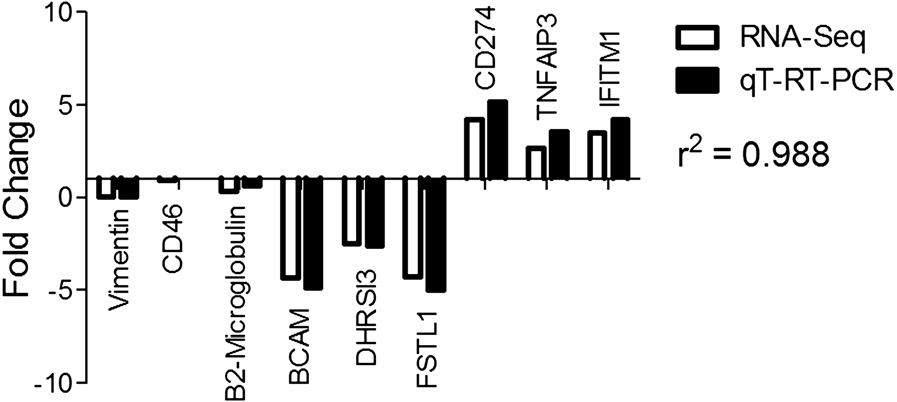
**qT-RT-PCR analysis of selected genes.** A total of nine host genes (3 downregulated, 3 not differentially expressed, and 3 upregulated) were selected for qT-RT-PCR validation. qT-RT-PCR was conducted in triplicate and the mean was graphed. The correlation coefficient (r^2^) was calculated using GraphPad Prism software.

**Figure 4:**
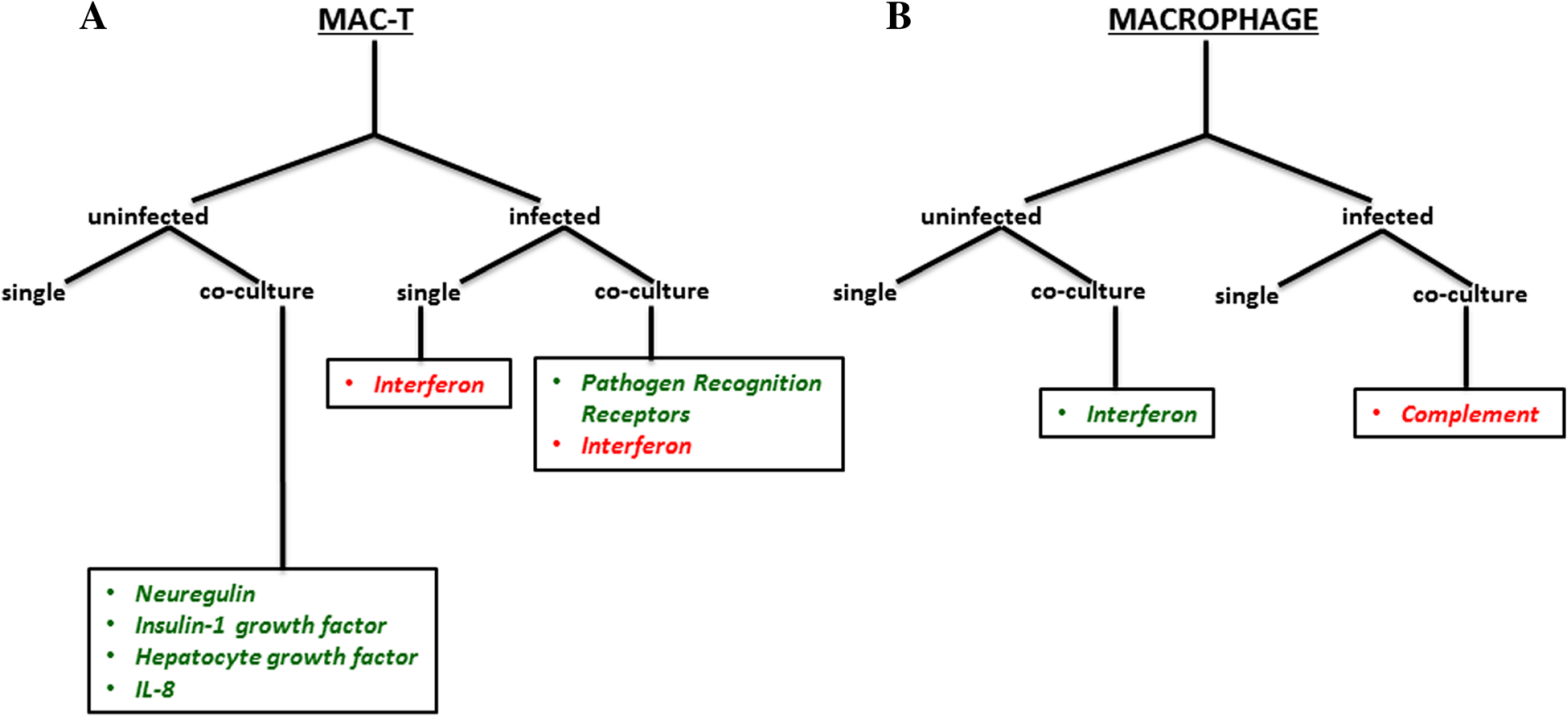
**Differentially regulated pathways in MAC-T cells and macrophages in response to co-culture and/or infection.** Differentially expressed host genes (P < 0.05) from **A)** MAC-T cells and **B)** macrophages were mapped to canonical pathways using the IPA knowledge base. Co-cultured cells were compared to cells cultured alone (uninfected and infected). Downregulated pathways are highlighted in green and upregulated pathways are highlighted in red.

Contrary to the long-held assumption of the epithelium acting as a bystander in microbial infection, we have shown that pathogen invasion at the epithelium interface is a dynamic process that results in macrophage recruitment to the infection site in an epithelium/macrophage co-culture system, which may aid in pathogen establishment and survival [33]. Macrophage recruitment is most likely influenced by the type of host cell MAP first encounters and extrinsic factors secreted during cell to cell cross-talk (eg. epithelium and macrophage). RNA-seq and Ingenuity Pathway Analysis (IPA) analyses identified 876 and 136 differentially expressed genes, respectively, that were either mapped to functional networks or to canonical pathways, in MAC-T cells grown under co-culture conditions in comparison to MAC-T cells cultured alone (Additional file 1: sheets 1 and 2 and Figure 2A). The 136 genes were categorized into cellular and molecular functions, which identified macrophage influence on functions related to cell growth and proliferation, cell development, protein synthesis, cellular movement and cell death (Figure 2A). Nine genes from the host (3 downregulated, 3 upregulated and 3 not differentially expressed) were selected for qT-RT-PCR validation (Figure 3). qT-RT-PCR fold changes were compared against those obtained from RNA-seq and the correlation coefficient (r^2^) was calculated (Figure 3). The r^2^ was 0.988, indicating a strong correlation between RNA-seq and qT-RT-PCR data (Figure 3). The majority of IPA mapped genes were downregulated in comparison to MAC-T cells cultured alone and were placed into networks involving 1) protein synthesis and cell cycle, 2) assembly and organization, cellular function and maintenance, nucleic acid metabolism, 3) cancer, dermatological diseases and conditions and lymphoid tissue structure and development and 4) cell cycle, cell morphology, and cellular assembly and organization (Additional files 2, 3, 4 and 5, respectively). Top canonical pathways (e.g. those with the most number of target molecules) were downregulated and encompassed neuregulin, insulin-1 growth factor (IGF-1), hepatocyte growth factor (HGF), and interleukin-8 (IL-8) signaling pathways (Figure 4). Downregulation of the listed canonical pathways are involved in the cessation of cell growth and inflammation. For example, activation of the neuregulin signaling pathway produces molecules that function as mitogens, differentiation agents and transforming agents in epithelial cells (Figure 4A) [35]. Also, the IGF-1 signaling pathway induces cell growth and survival via atypical protein kinase C (ApkC) (Figure 4A) [36]. Again, cell growth is also a major function of the HGF pathway (Figure 4A) [37]. The IL-8 signaling pathway, which promotes inflammation, is likely downregulated as tolerance has been achieved within MAC-T cells (Figure 4A) [38,39].

### Co-cultured macrophages are characterized by an immune tolerant phenotype reminiscent of subepithelium dome (SED) macrophages

Six-hundred two genes were differentially expressed between co-culture vs. macrophage alone by RNA-seq, and 77 of them were mapped by IPA (Table 1 and Additional file 1: sheet 3). Genes were categorized by biological and molecular functions, which included cellular function and maintenance, cellular movement, cell morphology, cell-to-cell communication, and antigen presentation (Figure 2A). Like co-cultured MAC-T cells, the majority of genes identified in macrophages under co-cultured conditions were downregulated compared to macrophages cultured alone. Downregulation of genes associated with antigen presentation suggest establishment of tolerance in macrophages [32,40]. This is further strengthened by decreased expression in 1) inflammatory disease, 2) molecular transport, and 3) infectious disease (Additional files 6, 7 and 8, respectively). In addition, inflammatory pathways, such as interferon signaling, were downregulated (Figure 4B). These data suggest that MAC-T cell extrinsic factors promote a tolerant phenotype that is characteristic of SED and lamina propria macrophages. Similar to macrophages co-cultured with MAC-T cells, laminia propria macrophages do not produce inflammatory cytokines such as IL-6, IL-1 and TNF-α. Tolerance is also achieved within MAC-T cells due to extrinisic factors released during co-culture with macrophages, which is reflective of the intestinal epithelium. These data suggest that the MAC-T cell/macrophage co-culture system may serve as an *in vitro* model for the intestinal epithelium to study MAP-host interactions. Future studies should include further characterizations of co-cultured macrophages including the presence of macrosialin (CD86) and response to TLR angonists.

**Table 1:** Host differential gene expression and comparisons

**Comparison**	**All genes**	**Upregulated**	**Downregulated**	**Causation**
Infected vs. Uninfected MAC-T cells	547	232	315	MAP infection ***(A)***
Infected vs. Uninfected Co-cultured MAC-T cells	806	370	436	MAP infection ***with*** extrinsic factors from macrophages ***(B)***
Infected vs. Uninfected Macrophages	488	342	146	MAP infection ***(C)***
Infected vs. Uninfected Co-cultured Macrophages	570	263	307	MAP infection ***with*** extrinsic factors from MAC-T cells ***(D)***
Infected Co-culture MAC-T cells vs. Infected MAC-T cells	695	594	101	MAP infection ***and*** extrinsic factors from macrophages ***(E)***
Infected Co-cultured Macrophages vs. Infected Macrophages	826	452	374	MAP infection ***and*** extrinsic factors from MAC-T cells ***(F)***
Infected Co-cultured MAC-T cells vs. Uninfected MAC-T cells	876	751	125	Extrinsic factors from macrophages ***(G)***
Infected Co-cultured Macrophages vs. Uninfected Macrophages	602	347	255	Extrinsic factors from MAC-T cells ***(H)*** and MAP infection ***(C)***

### MAC-T cells infected with MAP downregulate complement receptor and have enhanced interferon activity

Five hundred and forty-seven genes (179 genes mapped using IPA) were differentially expressed in MAC-T cells infected with MAP in contrast to 806 genes (52 genes mapped using IPA) found in infected MAC-T cells under co-cultured conditions (Table 1, Additional file 1: sheet 4 and Figure 2B). Separate biological functions used by infected co-cultured MAC-T cells included protein folding and post-translational modification in contrast to infected MAC-T cells alone which contained cell death function (Figure 2B). The majority of upregulated genes under co-cultured conditions were involved in cellular growth and proliferation (Additional file 9) and DNA replication, recombination and repair in individual cell type culture (Additional file 10). Canonical pathway analysis revealed genes involved in PRR and interferon signaling under co-culture (Figure 4A). PRR signaling was limited to the complement receptor, which was downregulated in response to MAP infection (Figure 4A). Several reports indicate that complement receptor is used by pathogenic mycobacteria to gain entry into host cells; however, these studies focused on mycobacteria-macrophage interaction only [41-43]. It is possible that MAP uses an alternative entry route as genes associated with complement were also not upregulated in infected MAC-T cells cultured alone when compared to the uninfected control. MAP may downregulate the complement receptor in MAC-T cells co-cultured with macrophages in order to avoid immune recognition and potential clearance, which may occur due to the increased gene activity in co-cultured MAC-T cells compared to single cell type culture (Table 1). As opposed to a pathogen driven mechanism, the host may downregulate the complement receptor in order to prevent MAP invasion. In order to determine which scenario is correct a study utilizing multiple complement blocking strategies will be necessary. A common host mechanism employed under co-culture and single cell type culture conditions during MAP infection is the upregulation of genes found in the interferon signaling pathway (Figure 4A). Infected co-cultured MAC-T cells upregulated 2′-5′-oligoadenylate (OAS-1), myxovirus resistance-1 (MX1) and interferon-induced protein with tetratricopeptide repeats 3 (IFIT3) (Figure 4A). MAP infected MAC-T cells alone also upregulated OAS-1, MX1 and IFIT3; however, the host cell had increased expression of Signal Transducers and Activators of Transcription (STAT1) and interferon regulatory factor 9 (IRF9) genes (Figure 4A). 2′5′- oligoadenylate synthestase 2 (OAS-2), a double stranded RNA (dsRNA) binding protein, is commonly associated with an antiviral response, which is characterized by downstream interferon (IFN) signaling. Like dsRNA viruses, MAP may capitalize on OAS-2 manipulation in order to evade the host innate immune response. For example, nuclear oligomerization domain 2 (NOD2), a cytoplasmic pathogen recognition receptor that detects muramyl dipeptide (MDP; a hydrolyzed product of bacterial peptidoglycan), binds to OAS-2 to enhance RNase-L, an antiviral endoribonuclease, activity and subsequent IFN production [44]. IFN production has been linked with control of mycobacterial dissemination [45,46]. Interestingly, NOD2 has recently been recognized as a PRR for RNA viruses and RNase-L has been reported to be involved in clearance of intracellular bacteria [47,48]. Dampening of the NOD2-OAS-2 pathway may represent a universal mechanism in intracellular pathogen survival by innate immune evasion. STAT-1 is also linked to IFN production and its impairment leads to disseminated disease [49]. Together these data suggest that initial infection is marked by a check and balance system in which MAP downregulates the complement receptor to prevent immune recognition and the host upregulates the interferon pathway to clear MAP.

### Co-cultured macrophages infected with MAP upregulate the complement receptor to promote MAP entrance

Four hundred and eighty-eight genes (169 genes mapped by IPA) were identified in MAP infected macrophages cultured alone versus 577 differentially expressed genes in MAP infected co-cultured macrophages (109 genes mapped in IPA) (Additional file 1: sheet 5 and Figure 2C). The majority of assigned biological functions were common to both culture conditions (Figure 2C). However, antigen presentation and cell morphology functions were restricted to co-cultured conditions while cellular movement was associated with single cell type culture (Figure 2C). The cell death network was mostly downregulated in co-cultured macrophages in response to MAP infection in contrast to upregulation of cell-to-cell signaling and interaction (Additional files 11 and 12, respectively). Unlike MAP infected co-cultured MAC-T cells, co-cultured macrophages upregulated the complement receptor, C1q, in response to MAP infection (Figure 4B). This suggests that once MAP orchestrates its exit from MAC-T cells, it preferentially utilizes the complement receptor for its own uptake into macrophages.

Liver X receptor/ Retinoid X receptor (LXR/RXR) activation pathway was identified in MAP infected macrophages cultured alone (Figure 4B). The LXR/RXR activation was shown to contribute to host protection against *M. tuberculosis* infection as determined by bacterial burden [50]. LXR/RXR protection may be due to its downstream proteins including nitric oxide synthetase-2 (NOS-2), interleukin 1-beta (IL-1β) and matrix metallopeptidase 9 (MMP9), which are all upregulated in infected macrophages (Figure 4B).

### MAP residing in MAC-T cells rebuilds its cell wall

**Figure 5:**
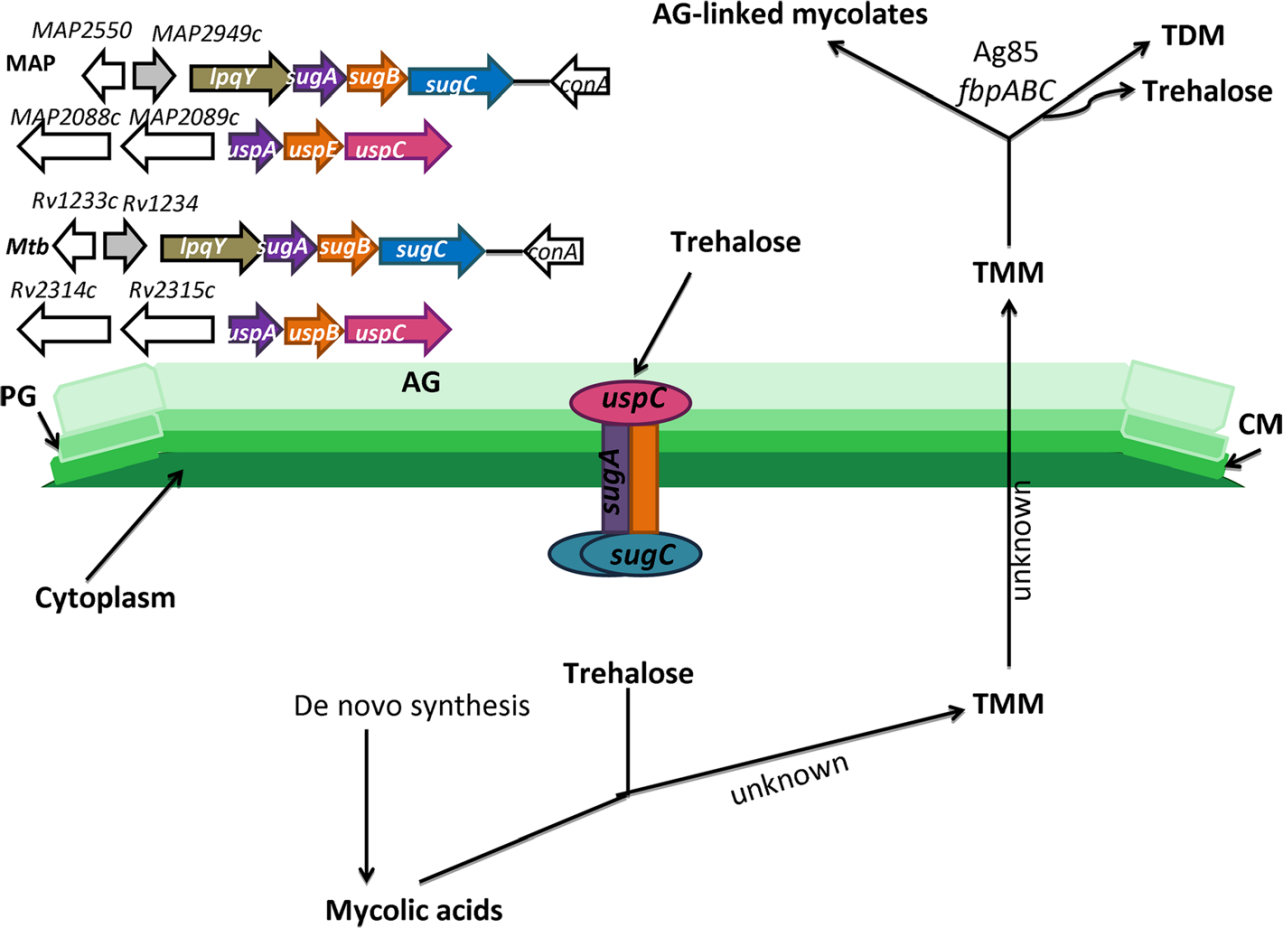
**MAP utilized the SugA-SugB-SugC pathway for trehalose recycling in MAC-T cells.** Differentially expressed MAP transcripts (P < 0.5) were categorized into networks using STRING ver. 9. Networks were cross-referenced against the Tuberculist, KEGG, and mycobacteria literature. Network analyses identified the upregulation of the SugA-SugB-SugC pathway in MAP inside MAC-T cells. Organization of the *sugABC* and uspABC operons in MAP and *M. tuberculosis* H37Rv (above). MAP UspA is a periplasmic sugar binding protein that forms a lipid anchor and binds to trehalose. Trehalose is transported into the MAP cell by a transmembrane protein (heterodimer of SugA and SugB) and SugC, a terminal ATP-binding cytoplasmic protein. It is hypothesized that trehalose interacts with mycolic acids by a currently unknown mechanism to produce TMM that is exported into the extracellular milieu to re-start the recycling process. *Abbreviations*: AG = arabinogalactan, PG = peptidoglycan, CM = cytoplasmic membrane, TMM = trehalose-containing molecules and TDM = trehalose dimycolate. Modified figure is based on the pathway/figure described by Kalscheuer et al. [58].

Comparisons of MAP transcripts during MAC-T cell infection to those in macrophage infection identified 448 differentially expressed genes (Additional file 1: sheet 6 and Table 2). Network analysis confirmed the presence of *sugA* and *sugC*, genes from an operon predicted to be involved in carbohydrate utilization from the host, and *uspC*, a lipid anchor associated with the *uspABC* operon that also plays a role in carbohydrate transport (Additional file 13) [51]. The *sugABC* operon, an ABC transporter, is best characterized in *M. tuberculosis* and is composed of a periplasmic sugar binding protein that forms a lipid anchor (LpqY), a transmembrane protein (a heterodimer of *sugA* and *sugB*) and a terminal ATP-binding cytoplasmic protein (*sugC*). It was previously thought that *sugABC* served to transport maltose or matodextrins; however, Edson had shown that both *M. tuberculosis* and *M. smegmatis* were incapable of growth when supplied with maltose as the sole carbon source [52-54]. The literature also indicates that host carbohydrate utilization by pathogenic mycobacteria is unlikely as the phagosome is carbohydrate poor and that host lipids provide the critical carbon and energy sources for growth [55,56]. It is likely that the stage of infection may determine carbohydrate availability within the phagosome. For example, Schnappinger et al. state that “there is no one phagosome” and transcriptomic profiles change concomitantly with time. In the study performed by Schnappinger et al., RNA was collected from intraphagosomal mycobacteria at 4, 24, and 48 h post infection as opposed to an earlier time point, such as this study, representing initial infection, which may impact carbohydrate availability [55]. Going beyond host carbohydrate metabolism, mycobacterial sugar transport systems have been implicated in virulence since transposon mutants of sugar transporters have a growth defect in macrophage and mice models [51,57]. Recently, Kalscheuer et al. has shown a novel role for LpqY-SugA-SugB-SugC in cell wall maintenance via disaccharide trehalose retrograde recycling [58]. Kalscheuer et al. demonstrated specific binding of LpqY to trehalose and subsequent uptake into the SugABC transporter. In the newly devised sugar transporter model, trehalose-containing molecules (TMM) are released during cell wall biosynthesis and are processed by mycolyl transferases from the antigen 85 complex, which result in the incorporation of the TMM mycolyl moiety to arabinogalacton (AG) for cell wall building. This process also yields the formation of trehalose dimycolate (TDM) and trehalose; trehalose is capable of binding onto LpqY, which initiates trehalose transport into the cell’s cytoplasm via SugABC [58]. Once inside the cytoplasm trehalose may interact with mycolic acids by an unknown mechanism to produce TMM that is exported into the extracellular milieu to re-start the cycle [58]. The authors suggest that trehalose recycling may be necessary to maintain sugars for mycolic acid biosynthesis and as an alternative carbon source [58]. We hypothesize that the MAP sugar transport system also serves a similar function as described for the *M. tuberculosis* LpqY-SugA-SugB-SugC pathway. Both MAP and *M. tuberculosis* operons are identically organized; however, our network lacks *lpq* and *sugB* (Figure 5). Although present in our MAP gene data sets, *sugB* was not considered for analysis as its p-value at 0.8 was not statistically significant (Additional file 1: sheet 6). As mentioned above, the MAP sugar transport network includes *uspC*, which serves the same function as *lpqY* in the *uspABC* operon. Protein BLAST comparisons show that UspC and LpqY are 67% identical, share bacterial extracellular solute-binding protein motifs, and have a predicted function as periplasmic solute binding lipoproteins. We suggest that *uspC* may also be used in the *sugABC* pathway as a TM binding protein (Figure 5). Further investigations into the MAP *sugABC* operon will elucidate the importance of trehalose-recycling in virulence and determine if UspC may functionally replace LpqY. Furthermore, *sugABC* may provide a novel therapeutic target for pathogenic mycobacteria as trehalose and trehalose uptake systems are not present in mammals, which may reduce drug side-effects that are commonly associated with current chemotherapeutics.

**Table 2:** MAP differential gene expression and comparisons

**Comparison**	**All genes**	**Upregulated**	**Downregulated**	**Causation**
Infected Co-Cultured MAC-T cells ***(7)*** vs. Uninfected MAC-T cells ***(3)***	215	125	90	Extrinsic factors from Macrophages ***(J)***
Infected Co-cultured Macrophages ***(8)*** vs. Uninfected Macrophages ***(4)***	15	13	2	Extrinsic factors from MAC-T cells ***(K)***

### Beta-oxidation is a universal MAP pathway in MAC-T cells and macrophages under co-culture and single cell type conditions

**Figure 6:**
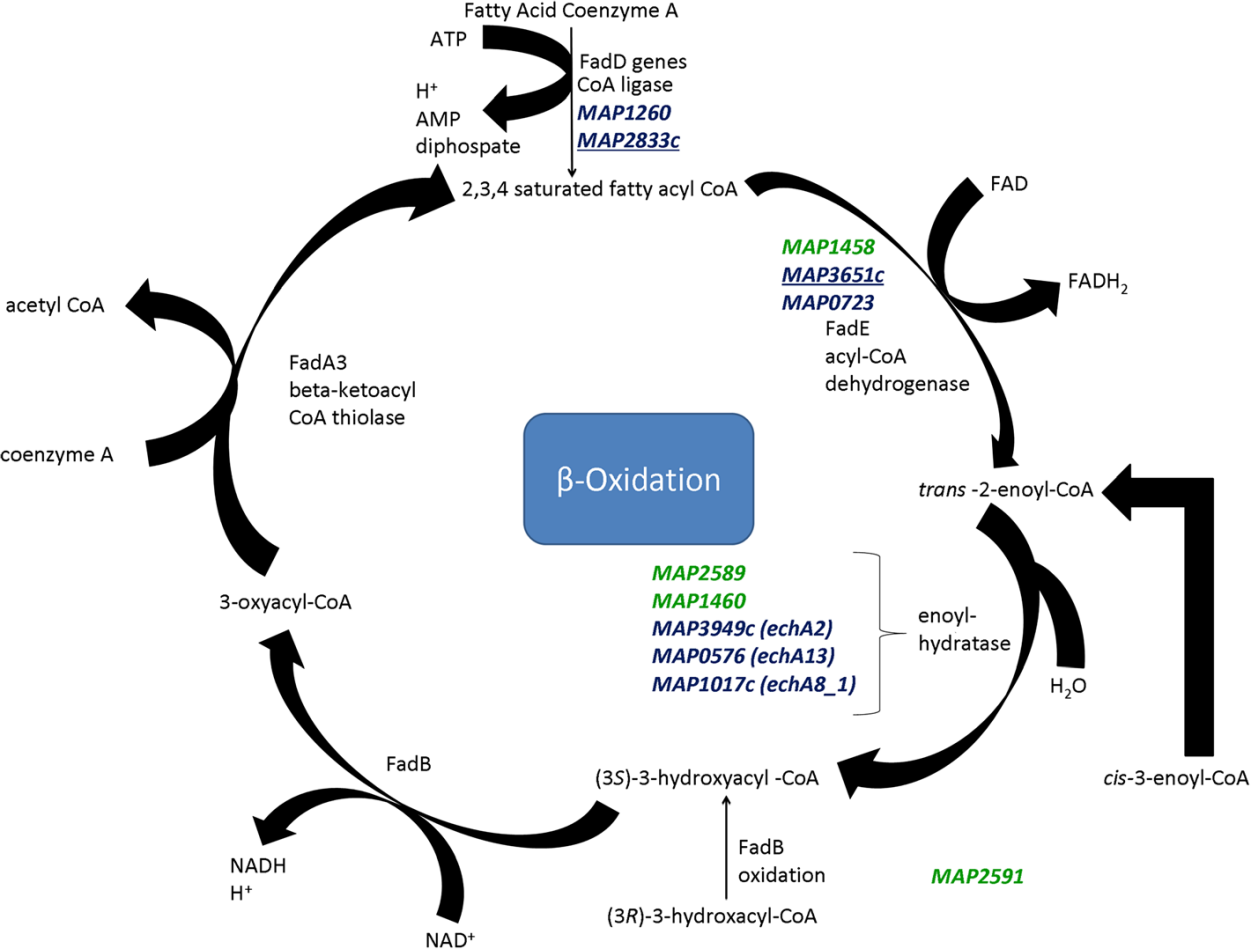
**MAP utilizes the β-oxidation pathway under all culture conditions.** MAP inside MAC-T cells (co-cultured and alone) and macrophages (cultured alone) upregulated genes involved in the β-oxidation pathway. Upregulated genes involved in β-oxidation were *fadD*, *fadE*, enoyl-hydratases, and a transcriptional regulator (*MAP2591*). Genes highlighted in dark blue were upregulated under single cell culture; underlined = macrophage, normal text = MAC-T. Genes highlighted in green were upregulated in MAC-T cells under co-cultured conditions.

Network comparisons against known MAP pathways found in KEGG mapped several MAP genes to the beta-oxidation pathway regardless of cell type and co-culture status (Additional files 13, 14 and 15 and Figure 6). The beta-oxidation pathway is utilized by mycobacteria to oxidize fatty acids as a sole carbon source in order produce acetyl-CoA, which enters into the Tricarboxylic Acid Cycle (TCA), to replicate [59,60]. *fadD* (CoA ligase), *fadE* (acyl-CoA dehydrogenase) and *echA* genes (enoyl hydratase) were found to be upregulated by MAP in MAC-T cells under single cell type culture conditions (Additional file 14 and Figure 6). The MAP profile in infected macrophages (single cell type infection) identified one *fadD* (*MAP2833c*) gene and one *fadE* gene (*MAP3651c*), indicating that beta-oxidation has just initiated within macrophages (Figure 6). Comparisons of infected co-cultured MAC-T cells against infected MAC-T cells, identified additional *fadE* gene (*MAP1458*), enoyl hydratases (*MAP2589* and *MAP1460*), and a transcriptional regulator (*MAP2591*) (Figure 6).

### MAP undergoes translesion synthesis and double stranded break repair to insure its survival within nitrosative MAC-T cells

**Figure 7:**
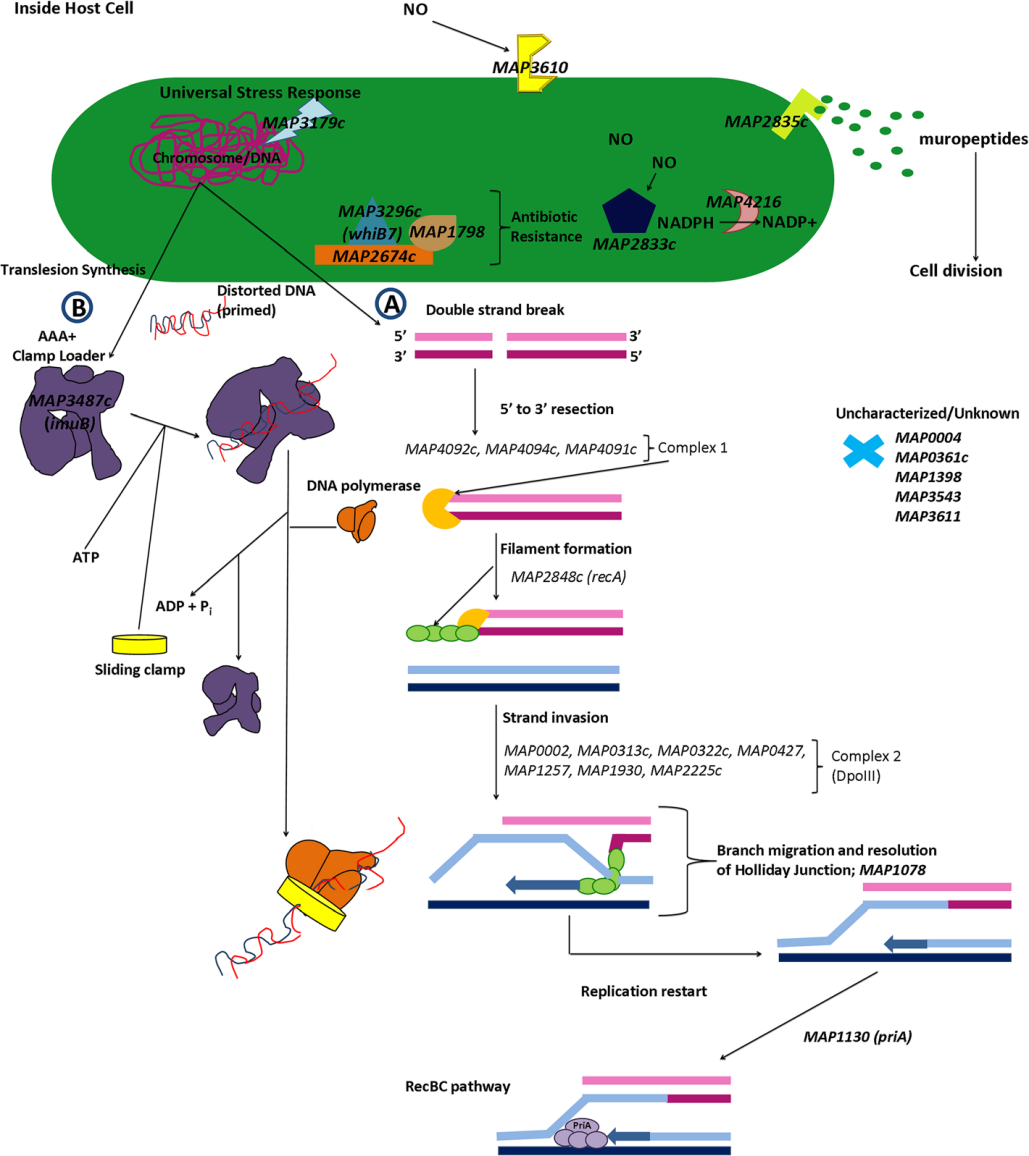
**MAP upregulates double strand break repair and translesion synthesis to resist the host immune response in MAC-T cells.** MAP responds to nitric oxide production inside MAC-T cells by upregulating a universal stress response gene, *MAP3179c*, which binds onto MAP DNA. In order to overcome NO, MAP upregulates *MAP4216* which causes a reducing environment inside the cell and subsequently turns on antibiotic resistant genes (*MAP1798*, *MAP2674c*, and *MAP3296c*. *MAP2835c*, a hydrolase, is involved in cell wall division and double strand break repair commences to correct any deleterious effects created by NO. NO may also cause irreparable damage to DNA bases; therefore, MAP also employs translesion synthesis to continue replication. This may also cause insertion mutations; the impact of this is currently unknown.

Two hundred and fifty-one differentially regulated MAP genes were uncovered in MAC-T co-cultured vs. macrophage co-cultured comparisons (Additional file 1: sheet 7). Further analysis identified a major network pertaining to MAP replication (Additional file 15). The first characterized network gene, *MAP3179c*, was determined to function as a universal stress response gene that when bound to chromosomal DNA, initiates the SOS response (Figure 7). Further examination of the network indicated that *MAP3179c* became activated due to nitric oxide sensing within the bacterial cell. (Figure 7) [61]. The presence of NO is suspected to result in DNA damage. *MAP2835c* has an upstream SOS binding box and like *MAP3179c* is activated due to nitric oxide buildup; therefore, DNA replication is likely inhibited by a yet to be identified mechanism until DNA repair can be accomplished [62]. MAP likely overcomes the stall in DNA replication by employing A) double strand break repair and B) translesion synthesis (Figure 7). Mapping of network genes onto the double strand break repair pathway for MAP within KEGG showed *MAP1078* and *MAP1130* involvement. MAP1078 is responsible for dissolving the Holliday junction after 5′ to ′3 resection and MAP1130 forms a primosome to resume DNA replication (Figure 7). It is possible that prolonged cell exposure to NO may cause some DNA bases to become irreparable [63]. Based on our network analysis this appears to be the case; therefore, MAP conceivably employs translesion synthesis (TLS) (Additional file 15). TLS is a damage tolerance pathway that allows a cell to replicate past DNA lesions and distortions by using an error-prone or lesion bypass polymerases [64]. This process is expected to follow the Tool belt hypothesis, which has been shown in *E. coli* and *M. tuberculosis*[65,66]*.* MAP3487c (referred to as ImuB; predicted Y-family polymerase) contains a β-clamp motif that enables recruitment and rapid interchange of replication machinery proteins pertaining to DNA repair and lesion bypass polymerases (sliding clamp) at the replication fork [66,67] (Figure 7). Along with resuming DNA replication, mutations will be inserted into the nascent strand. The implications of this are unclear. Nitric oxide may also interact with MAP2833c, which contains a nitrobindin binding domain that reversibly binds to nitric oxide, and cause further deleterious buildup within the cell [68]. The network implicates the involvement of *MAP4216*, a predicted glutathione reductase based on the presence of a pyridine nucleotide-disulphide oxidoreductase domain, as a source to defuse nitric oxide (Figure 7). Enzymes containing the pyridine nucleotide-disulphide oxidoreductase domain have been shown to protect cells from oxidative damage [69]. *MAP3296c*, also referred to as *whiB7*, is present within the network. WhiB7 from *M. tuberculosis* is involved in the transcription of antibiotic resistance programs including those for tetracycline, macrolide, lincosamide and aminoglycoside resistance [70-73]. In our model, WhiB7 is activated in the presence of a reducing environment caused by MAP4216. WhiB7 interacts with MAP1798, a potential antibiotic resistance protein, and MAP2674c, an antibiotic biosynthesis monooxygenase (Figure 7). Also, we show that *MAP2835c,* which contains a LysM (peptidoglycan binding domain) and closely resembles the cell wall hydrolase, *Rv2719c*, of *M. tuberculosis*, is upregulated. *MAP2835c* is hypothesized to have a role in maintenance of cell wall division despite the presence of DNA damaging agents [74-77]. These data suggest that MAP encounters a dynamic host environment that contains nitric oxide. MAP overcomes DNA damage and stops in replication by employing double strand break repair and TLS. This hostile environment also creates an opportunity for MAP to activate antibiotic resistance genes. This network has illustrated a number of potential targets for therapeutic design, including ImuB and WhiB7 (Figure 7). Further research involving mutation and deletion of the above genes will be required to validate the TLS pathway.

### MAP upregulates various transporters involved in lipid and nickel import in co-cultured MAC-T cells

**Figure 8:**
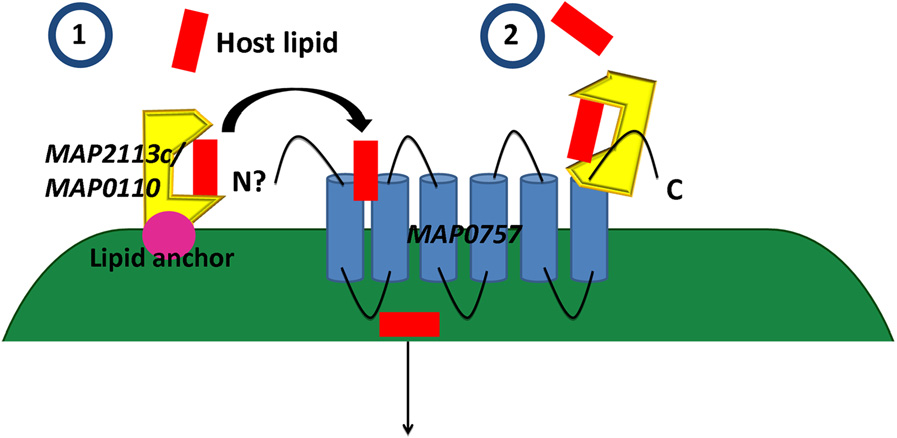
**MAP expresses mammalian cell entry genes to import host lipids in co-cultured MAC-T cells.** Network analysis identified upregulation of mce genes found in the *mce5*, *mce3* and *mce7* operons. *mce* genes are hypothesized to serve a lipid transport role as they are structurally similar to ABC transporters. MAP2113c and MAP0110 are predicted to be lipid binding proteins that may be 1) connected to a lipid anchor on the cell membrane or 2) inserted into the permease, MAP0757. Importation of lipids, potentially host derived, may be utilized for energy conversion.

Two networks were identified as being associated with mammalian cell entry (mce) and dipeptide/nickel transport (Additional file 15). Studies investigating *mce* gene function have linked *mce* expression with virulence and it is also believed that pathogenic mycobacteria upregulate *mce* family genes to gain entry into non-phagocytic cells [78-82]. However, *mce* genes are structurally similar to ABC transporters and are also thought to play a role in lipid or other molecule import into the cell [78,83-85]. The *mce* network is composed of *MAP0757* (*mce5*), an ABC transporter and permease, and *MAP2113c* and *MAP0110*, *mce* related genes that belong to the *mce3* and *mce7*[78]. The network suggests that although identified genes do not belong to the same operon that they may function together as a potential ABC import system. We hypothesize that MAP2113c and MAP0110 function as lipid binding domains and are either 1) tethered to the cell membrane via a lipid anchor or 2) inserts into MAP0757 (Figure 8). Once a lipid, possibly host derived, binds onto either MAP2113c or MAP0110 and is transferred to MAP0757 and is subsequently imported into the cell where it can be utilized for energy conversion (Figure 8).

**Figure 9:**
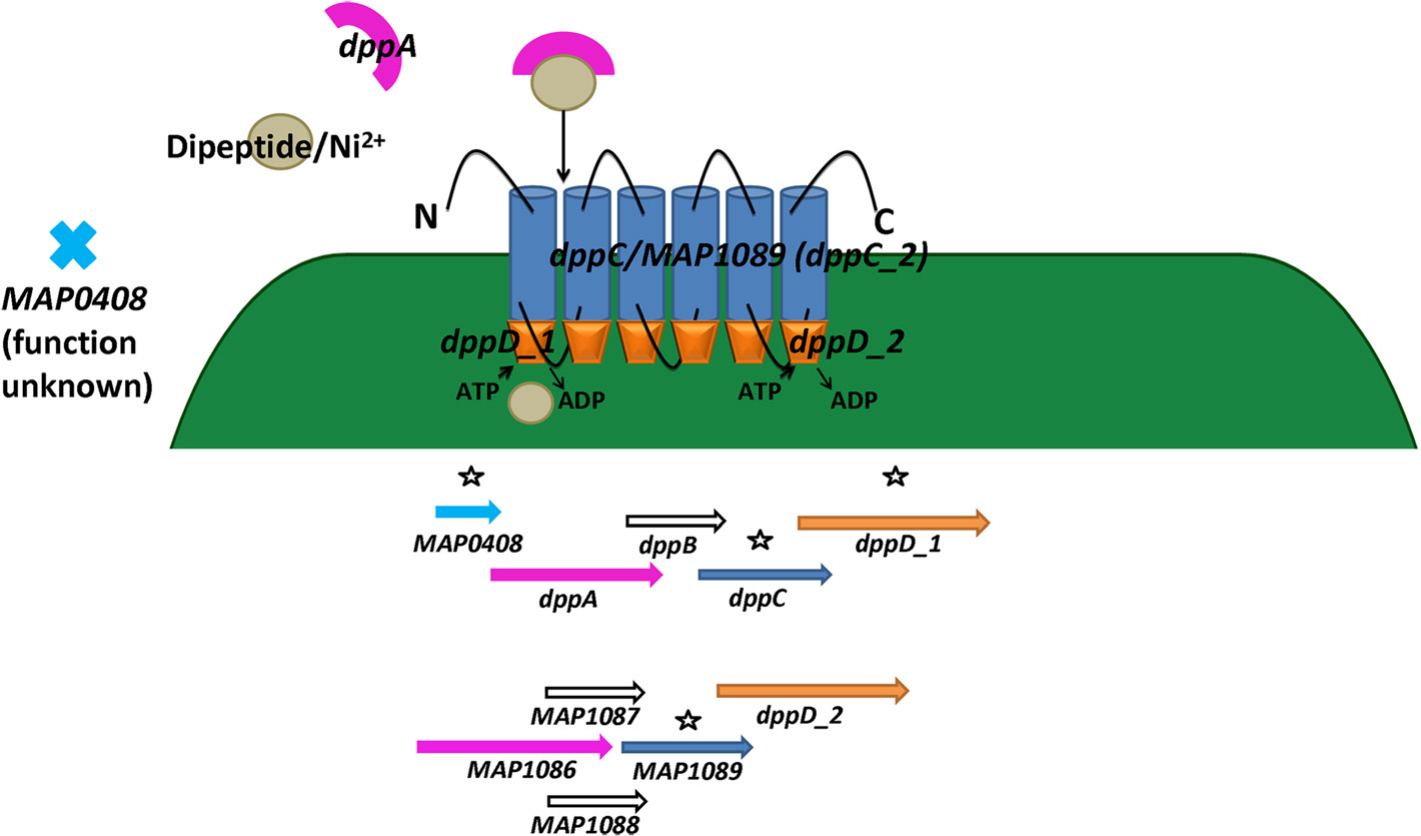
**MAP uses a nickel/dipeptide transporter inside co-cultured MAC-T cells.** An ABC transporter system is formed by MAP for nickel importation inside co-cultured MAC-T cells. DppA binds nickel/dipeptide and transports it into the cell using DppC, DppD_1, DppD_2, a permease system. The *dppABCD* operon (below). Genes identified in the network are noted with stars.

Nickel/dipeptide utilization is also accomplished through an ABC transporter system. In this model, dppA binds nickel/dipeptide in the extracellular milieu and transfers it to the permease, dppC (Figure 9). dppD_1 and dppD_2 represent terminal molecules in the system and drives nickel/dipeptide into the cell (Figure 9). Pathogenic mycobacteria have developed numerous systems for the importation and sensing of nickel as it is involved in cell growth and homeostasis [86,87].

### Defining the dance of seduction: the host-MAP interactome

In order to link host biological processes to pathogen responses, we examined all differentially expressed genes from host and pathogen (n = 1795) and conducted a K-clustering analysis to determine correlations in expression. The K-clustering analysis identified 81 host genes that shared a similar pattern profile with 77 MAP genes (Additional file 1: sheets 8 and 9 and Additional file 16). Host expression profiles were dependent upon co-culture status (Additional file 1: sheet 9). Host genes were categorized into Gene Ontology (GO) biological processes using DAVID software (Tables 3 and 4). The majority of GO processes upregulated in MAC-T cells were related to cell maintenance (e.g. cell division, cell cycle, DNA replication, etc.) while downregulated processes included the immune response and signal transduction regulation (e.g. kinase, phosphorylation and transferase activities) (Table 4 and Additional file 1: sheet 10). In contrast to differentially expressed GO biological process in MAC-T cells, macrophages showed upregulation of pathways involved in pathogen elimination and host preservation (e.g. defense, immune, inflammatory, and wound responses) and downregulation of immune responses primarily associated with 2′5′ oligoadenylate synthetase and major histocompatibility class 1 (MHCI) related proteins (Table 5 and Additional file 1: sheet 11). MAP genes identified by K-cluster analysis were organized into Cluster of Orthologous Groups (COG) (Table 5 and Additional file 1: sheet 8). As expected, the majority of differentially regulated genes were categorized as an unknown function (Table 5) due to the large number of hypothetical genes within the MAP K-10 genome [88]. Other functional groups of interest included defense mechanism, replication, recombination and repair, secondary metabolites biosynthesis, energy production and conversion, and lipid transport and metabolism (Table 5). COG identification indicates that initial infection is characterized by a pathogen program specific for MAP establishment within the host as defined by bacterial replication in face of strenuous host immune and defense responses.

**Table 3:** Differentially expressed Go biological processes in response to map infection inside MAC-T cells

	**GO process**	**Number of genes**	**P-Value**	**Benjamini score**
**Upregulated**	Cell cycle process	36	2.42E-12	2.43E-09
	M phase	2	7.38E-12	3.71E-09
	Nuclear division	15	8.97E-12	3.01E-09
	Mitosis	30	8.97E-12	3.01E-09
	Cell division	2	1.29E-11	3.24E-09
	M phase of mitotic cell cycle	15	1.81E-11	3.65E-09
	Organelle fission	30	2.15E-11	3.60E-09
	Cell cycle phase	18	2.36E-11	3.40E-09
	Cell cycle	8	4.14E-11	5.21E-09
	Mitotic cell cycle	16	1.17E-10	1.30E-08
	Microtubule-based process	12	3.14E-05	0.00315028
	DNA metabolic process	15	3.99E-05	0.003643606
	DNA replication	9	4.40E-05	0.003681565
	Microtubule-based movement	9	7.00E-05	0.00540001
	DNA replication initiation	4	1.80E-04	0.0128323
	Negative regulation of molecular function	8	5.69E-04	0.03743265
**Downregulated**	Immune response	19	1.06E-05	0.011956577
	Regulation of protein kinase activity	10	1.17E-04	0.064589297
	Regulation of kinase activity	10	1.76E-04	0.064494576
	Ear development	7	1.94E-04	0.053853857
	Regulation of phosphorylation	12	1.96E-04	0.043562316
	Regulation of transferase activity	9	2.27E-04	0.042105651
	Regulation of cell proliferation	15	2.64E-04	0.042029006
	Regulation of phosphorus metabolic process	12	2.89E-04	0.040342208
	Regulation of phosphate metabolic process	12	2.89E-04	0.040342208
	Negative regulation of protein kinase activity	6	3.44E-04	0.042649138

**Table 4:** Differentially expressed GO biological processes in response to MAP infection inside macrophages

	**GO process**	**Number of genes**	**P-Value**	**Benjamini score**
**Upregulated**	Defense response	18	1.10E-07	1.46E-04
	Inflammatory response	12	8.84E-07	5.84E-04
	Immune response	19	1.14E-06	5.04E-04
	Response to wounding	14	5.02E-06	0.001656
	Positive regulation of cellular component organization	9	5.07E-06	0.001341
	Regulation of cell adhesion	9	7.37E-06	0.001623
	Positive regulation of cell adhesion	7	8.82E-06	0.001664
	Positive regulation of organelle organization	6	3.53E-05	0.005815
	Lipid biosynthetic process	12	9.28E-05	0.013545
	Positive regulation of cell motion	6	2.03E-04	0.026443
	Fatty acid biosynthetic process	7	2.52E-04	0.029821
	Regulation of cytokine biosynthetic process	6	3.47E-04	0.037516
	Acute inflammatory response	6	6.23E-04	0.061455
**Downregulated**	Immune response	12	4.92E-04	0.03621235

**Table 5:** COG function

**Function**	**Number of genes**
Defense mechanism (V)	1
Secondary metabolites synthesis (Q)	1
Energy production and conversion (C)	1
Replication, recombination and repair (I)	5
Lipid transport and metabolism (I)	5
Function unknown (S)	60

The 77 MAP genes constitute two significant networks (Additional file 17). Network 1 is composed of 3 upregulated genes, *MAP3980*, *MAP1913c*, and *MAP2495*, in MAC-T cells and macrophages (Additional file 17). Predicted functions involve sensory transduction regulation (*MAP3980*) and endonuclease activity for homing activity (*MAP2495*). *MAP1913c* does not contain any known structural motifs and is declared as a gene of unknown function. *MAP3980* resembles *ybjN*, a sensory transduction regulator and orphan gene found in *Escherichia coli*[89,90]. *ybjN* has been show to play a role in cell motility, aggregation, exopolysaccharide production, and biofilm production [89]. These functions are dependent upon *ybjN* expression as overexpression of *ybjN* reduced the above processes [89]. However, upregulation of *ybjN* activated the SOS response pathway [89]. Homing endonucleases, like *MAP2495*, are highly-specific DNA cleaving enzymes that initiate double-stranded break repair [91,92]. Increased expression of *MAP3980* and *MAP2495* indicates a hostile host environment characterized by oxidative and nitrosative stresses, which is further supported by double-stranded break repair via *MAP1078* and *MAP1130* as well as translesion repair synthesis described in the MAC-T cell comparison to macrophages under co-culture conditions (Figure 7). This mechanism is most likely used as a survival strategy for pathogen genome stability in face of a strenuous host response [66,93-95].

**Figure 10:**
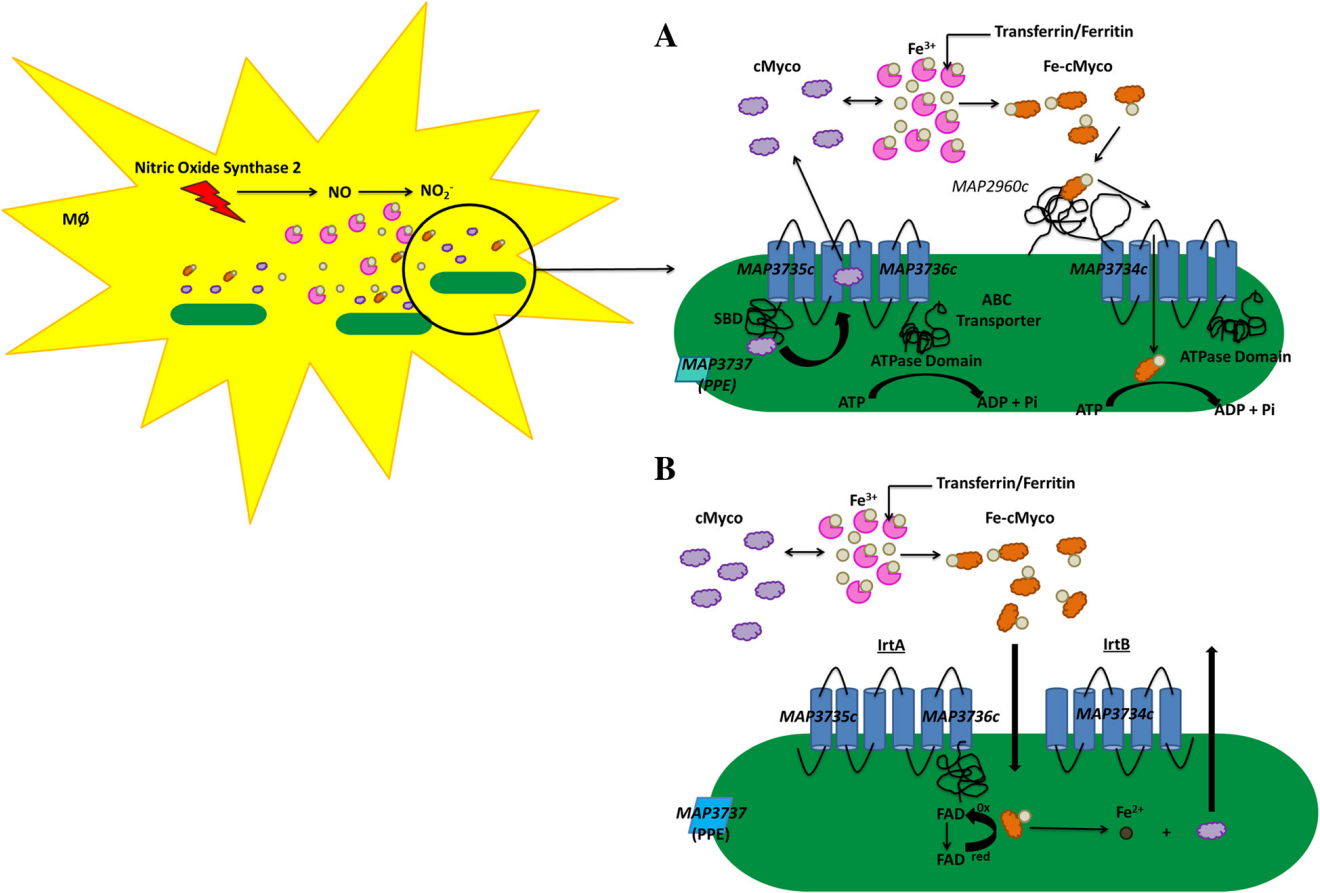
**MAP upregulates a novel iron pathway, *irtAB*, for iron assimilation in response to host nitric oxide synthase-2 expression.** Interactome network analysis identified a network composed of 4 genes (*MAP3734c-3736c* and *MAP3737*). *MAP3734c-3736c* forms an iron regulation pathway and is the equivalent of *irtAB* in *M. tuberculosis* H37Rv. **A)** IrtA (MAP3735c and MAP3736c) binds and releases non-ferrated carboxymycobactin (cMyco) into the host cell. cMyco binds iron (Fe-cMyco) and is transported back into MAP via MAP2960c (not identified in interactome) and IrtB (MAP3734c). MAP3737, a PPE protein, may serve as a signal transduction molecule and sense NO buildup. Upregulation of this pathway is linked to NOS-2 expression by the host. Figure is based in part by Farhana et al. [106]. **B)***MAP3734c-3736c* forms a homodimer and transport Fe-cMyco inside the MAP cell. Once Fe-cMyco is inside, the FAD-binding domain of IrtA (MAP3735c and MAP3736c) becomes activated and binds to flavin, which accepts electrons from NAD(P)H. This allows flavin reductase to function as an iron reductase. Reduction of Fe^3+^ to Fe^2+^ dissociates iron from carboxymycobatin so it can be utilized by MAP.

Network 2 was composed of an operon (*MAP3734c-3736c*) and a neighboring gene (*MAP3737*) that were transcribed in opposite directions and suspected in iron regulation (Additional file 17). Iron is utilized by intracellular bacteria in redox reactions, electron transport, replication and other essential functions [96-98]. Due to iron limitation within the host (e.g. iron bound to transferrin, lactoferrin or ferritin), pathogenic mycobacteria have developed several strategies for iron sequestration and storage [97-100]. A critical iron acquisition mechanism is the synthesis and transport of siderophores (low-molecular-weight Fe^+3^ chelators), which include mycobactin (cell wall associated) and carboxymycobactin (secreted) [101,102]. All genes in network 2 were found to be upregulated in both cell types (Additional file 17). However, co-cultured macrophages showed a decrease in transcription compared to macrophages cultured alone. Reduced MAP transcriptional activity within co-cultured macrophages may be due to dampening host responses caused by MAC-T cell extrinsic factors. *MAP3734c-3736c* and *MAP3737* are designated as hypothetical genes; however, domain and motif searches determined that *MAP3734c*- *3736c* form an ABC-type multidrug transport system and *MAP3737* has a Proline-Proline -Glutamate (PPE) domain. *MAP3734c*-*3736c* belong to the large sequence polymorphism 14 (LSP14) and contain nucleotide binding Walker A (WA), Walker B (WB), and ABC transporter Signature Motifs (SM) at the C-terminal end [103]. Also, *MAP3735c* has an N-terminal Siderophore Binding Domain (SBD) that resembles substrate binding domains of siderophore uptake systems, such as the ferric enterobactin transport ATP-binding protein, FepC [54,104]. Furthermore, *MAP3735c* and *MAP3736c* are predicted to form a heterodimer that is composed of 6 transmembrane segments in contrast to *MAP3734c* that forms 5 transmembrane segments. BLAST comparisons and reference to the Tuberculist database server (http://genolist.pasteur.fr/TubercuList/) listed *Rv1348* (*MAP3735c* and *MAP3736c* have 42 and 40 per cent amino acid identity, respectively) and *Rv1349* (*MAP3734c* is 34 per cent identical at the amino acid level) as orthologs to *MAP3734c-MAP3736c*. *Rv1348* and *Rv1349* were first reported to be repressed in the presence of iron by the direct regulation of the Iron dependent Regulator (*ideR*) and later identified as an ABC transport system involved in iron acquisition [99,105]. Rodriguez and Smith showed that mutants of *Rv1348* and *Rv1349* (also referred to as iron-regulated transporters (Irt) A and B, respectively) failed to replicate efficiently *in vitro* under iron-deficient conditions, in THP-1 cells, and inside the lungs of C57/B6 mice [105]. Rodriguez and Smith reasoned that IrtA and B may be involved in mycobactin synthesis; however, the *irtA* mutant and *irtAB* double mutant strains produced equivalent amounts of mycobactin compared to the wild-type strain [105]. Although *irtAB* was determined not to be involved in mycobactin synthesis, the growth defect was linked to the inability of the *irtAB* mutant to utilize iron bound carboxymycobactin (Fe-cMyco) [105]. *irtA* and *irtB* involvement in Fe-cMyco utilization is further supported by a study conducted by Farhana et al., which elucidated the *irtAB* iron trafficking mechanism utilizing recombinant IrtA and IrtB packaged into liposomes and *irtA* and *irtB* knock out mutants in *M. smegmatis*[106]. According to the model proposed by Farhana et al., IrtAB functions as a novel carboxymycobactin cytoplasmic exporter-importer system, in which newly synthesized, non-ferrated carboxymycobactin binds specifically onto the IrtA SBD and is exported into the extracellular milieu to sequester Fe^3+^[106]. Farhana et al. show that Fe-cMyco binds to an IdeR independent siderophore interacting protein, *Rv2895c*, found on the cytoplasmic membrane that forms a two-component importer with IrtB [106]. In addition to Fe-cMyco utilization, the IrtAB carboxymycobactin importer-exporter system may prevent toxic siderophore buildup inside the bacterial cell. Based on the Farhana model, *MAP3734c*-*MAP3736c* functions as a carboxymycobactin importer-exporter system that is activated due to iron limitation within the host (Figure 10A). *MAP3734c* forms a heterodimer with *MAP3735c* and exports non-ferrated carboxymycobactin into the extracellular milleu, where the siderophore may sequester iron. Fe-cMyco is transported back into the cell by *MAP3736c* and is subsequently used for processes requiring iron (Figure 10A). It is important to note that we did not find *MAP2960c*, the equivalent of *Rv2895c*, within network 2 nor in the listed MAP interactome genes. Recently the Farhana model has been brought into question due to the exclusion of confirmatory experiments such as, testing recombinant IrtB proteoliposomes for siderophore export and recombinant IrtA proteoliposomes for siderophore import, characterization of protein topology within liposomes, and creation of *irtA* and *irtB* knock out mutants in *M. tuberculosis*[107]. Rather than functioning as a carboxymycobactin importer-exporter system, Ryndak and others propose that IrtAB forms one ABC transporter necessary for Fe-carboxymycobactin and iron assimilation via ferric iron reduction [107]. In the study performed by Ryndak et al., the amino terminus domain was shown to contain a FAD-binding domain and when mutated abrogated IrtAB function and iron utilization [107]. The authors suggest that the Farhana model may be reflective of *M. smegmatis* only and has limited translation to *M. tuberculosis*[107]*.* Furthermore, Ryndak et al. state that the positive chrome azure S (CAS) assay from Farhana et al. is most likely due to exochelin, which composes the majority of secreted siderophores by *M. smegmatis*, and not Fe-carboxymycobactin [107]. Under the Ryndak model, MAP3734c-MAP3736c forms a single ABC transporter and the IrtA FAD binding domain functions after Fe-carboxymycobactin translocation (Figure 10B). The FAD binding domain of IrtA (MAP3735c and MAP3736c) binds flavin, which is reduced by NAD(P)H and dissociates and serves as a ferric reductase and is sustained by a “ping-pong” mechanism (accepting electrons from NAD(P)H and then reducing complexed Fe^3+^) (Figure 10B). Due to study design concerns from the Farhana et al. model and failure to identify *MAP2960c*, we propose that the correct iron assimilation pathway for MAP is likely the Ryndak et al. model. In addition to *MAP3734c*-*MAP3736c*, we have identified *MAP3737*, a PPE related gene, within the iron network (Additional file 17). Research performed by Rodriguez et al. showed that genes from the PE/PPE family are upregulated during iron limitation and repressed by IdeR in *M. tuberculosis*[99]*.* Structural studies by Strong et al. suggest the PPE proteins may serve as signal transduction molecules as they resemble the cytoplasmic domain of the serine chemotaxis domain (Tsr) [108]. The Tsr cytoplasmic domain functions as a multidomain protein capable of sensing extracellular signals, which are transmitted into the cell via a phosphorylation cascade [108]. Furthermore, we have identified upregulation of nitric oxide synthtase 2 (NOS-2) by host cells, which created a nitrosative environment that has been shown to enhance iron-scavenging programs in *M. tuberculosis*[55]. Upregulation of NOS-2 may promote *MAP3734c-3736c* expression due to *MAP3737* sensing, which would activate the iron uptake system (Figure 10A and B). The *MAP3734c*-*3736c* iron assimilation models (Farhana and Ryndak) argue that mycobactin is not required for iron uptake from Fe-cMyco. This is further strengthened by the growth of *M. tuberculosis mbtB* mutant controls, which do not synthesize mycobactin or carboxymycobactin but have an intact *irtAB* system, when provided with exogenous Fe-cMyco [105]. This may also explain successful MAP replication inside macrophages and mycobactin independence in some MAP laboratory strains cultured *in vitro* (provided inclusion of 1% ferric ammonium citrate and pH ~ 5.5) despite a truncation in the EntE domain of, the first gene in the mycobactin biosynthesis gene cluster [88,103,109-111]. It is also important to note that *in vitro* cultivation of isolated MAP from feces and tissue samples require medium supplementation by mycobactin; therefore, the possibility remains that *MAP3734c*-*3736c* activity may depend upon cues provided by the host cell, such as nitric oxide, or multiple *in vitro* passages within the laboratory [112]. Similarly, *MAP3734c*-*3736c* may also be employed during a specific stage in infection as iron from Fe-cMyco transfer to mycobactins in the cell surface has been reported, albeit in an *in vitro* setting only [113]. This model contradicts the hypothesized loss of function of *mbtA* (based on genome sequencing) described by Li et al.. However, it is important to note that the consequences of the EntE truncation in *mbtA* have not been validated by functional studies, including those replacing the *mbtA* gene with a completed version. Interestingly, Barclay et al. have described mycobactin independence (e.g. does not require mycobactin supplementation) and subsequent mycobactin synthesis in MAP and *M. avium* after multiple rounds of in vitro subculture [114]. Barclay et al. showed that the synthesized mycobactin was structurally different than supplemented mycobactin using high-pressure liquid chromatography [114]. The potential remains that the *mbtA* truncation results in reduced function rather than an entire loss of function. We propose that *MAP3734c*-*3736c* and *MAP3737* may play an important role during initial infection as a previous study conducted by our group reported the downregulation of this operon in intestinal tissues collected from subclinical JD positive cows [28]. The discrepancy between the two studies is likely reflective of infection stage as the MAP transcriptome from tissues were found largely to be downregulated, which is characteristic of a “hunkered-down” phase associated with subclinical/latent infection as opposed to initial infection where multiple genes are employed for pathogen establishment [115]. Creation of *MAP3734c*-*MAP3736c* deletions will be necessary to assign a definitive role to this operon and which model best represents this operon as well as determine if infection stage may impact its activity. To our knowledge, this is the first study to show *irtAB* (*MAP3734c-3736c*) employment by MAP as well as in a co-culture model of infection. These data provide further support that *irtAB* is a functional iron assimilation system used by slow-growing mycobacteria during infection.

## Conclusions

We established a primary macrophage cell culture reminiscent of macrophages found within the lamina propria. We profiled both host and MAP transcriptomes simultaneously using massively parallel ultradeep sequencing technology. Contrary to the reported literature, MAP infection inside the epithelium is a dynamic process that involves immune dysregulation (e.g. downregulation of complement and increased interferon activity) as well as mycolic acid building and DNA repair by MAP. We propose that the epithelium serves as a pathogen “boot camp” that trains MAP for efficient long-term survival within the macrophage phagosome. This epithelium “boot camp” for MAP has for the first time been shown as an interactome of initial MAP infection. We have uncovered an iron assimilation system linked to NOS-2 activity by the host, which is unrecognized in MAP research. Creation of the interactome has created previously unknown MAP pathways and identified multiple targets for potential therapeutics as well as data that can be subsequently used for hypothesis-driven research.

## Methods

### Ethics statement

All animal work was conducted in accordance with the recommendations in the institutional guidelines and approved animal care and use committee (IACUC) protocols at the University of Minnesota (approval number 1106A01161). All other experiments were carried out in accordance with the University of Minnesota’s Institutional Biosafety Committee (IBC) approved protocol number 0806H36901.

### Bacterial cell culture

A stock culture of *Mycobacterium avium* subsp. *paratuberculosis* strain K-10 (MAP K-10) was maintained in Middlebrook 7H9 medium supplemented with 1% glycerol, 10% oleic-acid-dextrose-catalase (OADC), and mycobactin J (2.0 mg/L) (Allied Monitor, Fayette, MO) at 37°C with shaking at 120 rpm. Prior to co-culture invasion assays, MAP K-10 was subcultured at 1/10th the original culture volume until logarithmic growth was achieved (O.D._600_ = 1.0, 1 × 10^9^ CFU/mL).

### Mammalian cell culture

Monocyte derived macrophages (MDMs) were elutriated and matured separately from three Johne’s disease free dairy cows 2170 and 3210 as described. Briefly, blood was collected from the jugular vein into a chemically sterilized container containing 200 mL of heparin, which served as an anticoagulant. Blood was transferred into DNase/RNase free polystyrene conical tubes and centrifuged at 2, 200 rpm for 20 min at room temperature (RT). Buffy coats were collected, washed in 1X Dulbecco’s phosphate buffer saline (D-PBS), and layered on top of a 58% percoll gradient (Sigma-Aldrich, St. Louis, MO). The buffy coat-percoll layers were centrifuged at 3,000 rpm for 30 min and decelerated without brake application. Cells were removed from percoll, washed 3× in 1X D-PBS, and matured in Teflon flasks containing RPMI 1640 with 20% autologous serum for 4 d at 37°C in a humidified chamber containing 5% CO_2_.

MAC-T cells, derived from a bovine mammary epithelial cell line, were cultured in DMEM containing 10% fetal bovine serum at 37°C in a humidified chamber containing 5% CO_2_. Upon 90% confluence, cells were divided using TrypLE Express (Invitrogen, Carlsbad, CA) per manufacturer’s instructions. MAC-T cells were utilized in this study as this cell-line is considered a surrogate for the intestinal epithelium and the source of the cell line, the mammary, is hypothesized to serve as a reservoir for MAP in vivo [23].

### Co-culture invasion assay

All incubation steps involving the co-culture invasion assay occurred in a 37°C humidified chamber containing 5% CO_2_ unless otherwise stated. MAC-T/bovine MDMs co-culture invasion assays were conducted using a described method with slight modifications [33]. In short, approximately 2.0 × 10^4^ MAC-T cells were seeded on the apical side of 3.0 μm pore size transwell (12 well plate format) (Corning, Lowell, MA) and incubated in DMEM with 10% FBS for 4 d at 37°C. Once MAC-T cells were 70% confluent, bovine MDMs were seeded at ~ 2.0 × 10^4^ cells in the basolateral chamber and allowed to adhere for 2 h at 37°C. Upon completion of incubation, cells were gently washed 3× using 1X D-PBS to remove non-adherent cells and DMEM medium containing 10% FBS was replaced. MAC-T/bovine MDMs were incubated an additional 2 d to establish a link between cell types.

MAP K-10 was subcultured at 1/10th the original culture volume and incubated until an O.D._600_ of 1.0 was obtained. Upon optical density measurement, MAP K-10 was pelleted at 3,000 × g for 10 min, washed 3× in 1X D-PBS and resuspended in DMEM containing 10% FBS such that a MOI of 10:1 was achieved. In order to ensure a single cell suspension, resuspended MAP K-10 was vortexed for 5 min and repeatedly drawn through a 21 gauge needle syringe. The MAP K-10 suspension was incubated in a 37°C water bath for 5 min to sediment any remaining clumps. The upper 2/3 of the MAP K-10 suspension was used for the invasion assay. MAC-T cells were infected for 3 h, washed 3× in 1X D-PBS and allowed to recover for 30 min in DMEM containing 10% FBS. MAC-T cells were further washed as before and transwells were removed from the supports and transferred to a new sterile 12 well plate. Five hundred μL of TRIzol (Invitrogen, Carlsbad, CA) was separately mixed into each transwell and well from the original plate containing bovine MDMs and incubated for 5 min at room temperature to ensure complete lysis. All cell lysates were collected and stored at -80°C in individual RNase/DNase free 1.7 mL eppendorf tubes until further processing.

### Total RNA extraction

Prior to RNA extraction, work surfaces and equipment were treated with RNase Away (Molecular Bioproducts, San Diego, CA). RNA was extracted from twelve samples composed of 4 uninfected host cell types (Macrophage and MAC-T under single cell type or co-cultured conditions), 4 infected host cell types (Macrophage and MAC-T under single cell type or co-cultured conditions), 2 pathogen samples from macrophages and MAC-T cells under single cell type culture, and 2 pathogen samples from co-cultured macrophages and MAC-T cells (Figure 1). All samples were collected after 30 min p.i.. Cell lysates in TRIzol were mixed with sterile RNase-free 0.1 mm silica zirconium beads (Biospec) and homogenized using a MagNa Lyser for 4 min (Roche, Indianapolis, IN). RNA was extracted following manufacturer’s instructions (Invitrogen, Carlsbad, CA) and treated with Turbo DNase (Ambion, Austin, TX) at 37°C for 30 min. Turbo DNase was inactivated using phenol/chloroform extraction. RNA purity was assessed by measuring the 260/280 ratio with the Nanodrop ND-1000 (Nanodrop Products, Willimington, DE). Also, a direct PCR confirmed the absence of *β-actin* amplification in RNA samples. RNA samples were stored in 10 μL aliquots at -80°C until further processing.

### Enrichment of MAP RNA

Approximately 10 μL of RNA (one from each MAP treatment) was submitted to enrichment and amplification for MAP transcripts using MICROBEnrich (Ambion, Austin, TX) and MessageAmpII bacteria kit (Ambion, Austin, TX) per manufacturer’s instructions. Successful elimination of host RNA was determined by the RNA 6000 LabChip kit and Agilent Bioanalyzer (Caliper Technologies Corp., Hopkinton, MA and Agilent Technologies, Santa Clara, CA).

### Sample processing and RNA-Seq

Ten μg of RNA from each sample was suspended in 50 μL of nuclease free water. In order to ensure RNA integrity and purity, all samples were quantified using the RiboGreen assay per manufacturer’s instructions (Invitrogen, Carlsbad, CA) and analyzed on the Agilent Nanochip (Agilent Technologies, Santa Clara, CA). RNA samples were required to have a RNA Integrity Number (RIN) of 8 or greater to proceed with library creation. The RNA-Seq library was created using the mRNA Seq library preparation kit per manufacturer’s instructions (Illumina Inc., San Diego, CA). The purified library was later validated and quantified using the Agilent High Sensitivity Chip (Agilent Technologies, Santa Clara, CA), picogreen assay (Invitrogen, Carlsbad, CA), and KAPA qPCR (KAPA Biosystems, Woburn, MA) as described by the corresponding manufacturer. The Illumina cBOT (Illumina Inc., San Diego, CA) was used for cluster generation. Briefly, the template (samples in 8-plex) was immobilized to a random oligo lawn on the flow cell surface, which was later amplified, linearized, blocked and hybridized to the sequencing primer. The clustered flow cell was then transferred and loaded into the Illumina Genome Analyzer II_x_ (GaIIx) (Illumina Inc., San Diego, CA). Sequencing was conducted in pair-end reads. Twenty million reads (200 bp insert size) and 7.5 million reads (150 bp insert size) were recorded for each bovine and MAP samples, respectively. A Pass/Fail score was calculated for all samples using Consensus Assessment of Sequence and Variation (CASAVA) Version 1.6 software (Illumina Inc., San Diego, CA) based on reads quality score (Additional file 1: sheets 1 and 12). Average and individual reads had Phred (passRead) scores of above 30 (Additional file 1: sheets 1 and 12 and Additional file 18A and C). All sequence information was converted into FASTQ files for each sample. All FASTQ files are available through NCBI SRA (http://www.ncbi.nlm.nih.gov/bioproject/218473), Project ID PRJNA218473.

### RNA-Seq mapping to reference genomes

Bovine and MAP read files were analyzed in Galaxy (https://sites.google.com/a/umn.edu/galaxy-umn/home) using Tuxedo Suite Tools. The sequence quality was checked and the low quality 5′ end bases were trimmed. Bovine FASTQ files were mapped to the *B. taurus* genome (Btau 4.0) using TopHat (v2.02) with 2 mismatch setting. The differential gene expression (DGE) was analyzed using Cufflinks (Cuffdiff program) [116-118]. MAP FASTQ files were mapped to the MAP K-10 genome using Bowtie with 2 mismatch and the DGE was examined using edgeR program in BioConductor. DGE was determined by a q-value cutoff of 0.05 or p-value cutoff of 0.05 as determined by Cuffdiff and edgeR, respectively. Principal component analysis (PCA) in Expressionist using reads per kilobase of exon model per million mapped reads (RPKM) and fragments per kilobase of exon model per million mapped reads (FPKM) values was conducted in order to insure sample stratification (Additional file 18B and D). As expected, host samples were stratified by cell type and MAP samples were separated by infected cell type (Additional file 18B and D).

### Host canonical pathway and functional network analyses

Bovine transcripts and expression values identified by DGE were uploaded into Ingenuity Pathway Analysis (IPA) (Redwood City, CA) and screened for canonical pathways within the IPA library and knowledge base. The IPA library consists of pathways from the mouse, human and rat genomes; therefore, identified pathways were based on homologous genes from the bovine genome. Genes with a q-value cutoff of 0.05 were considered for pathway analysis.

Bovine transcripts and corresponding expression levels were mapped to gene objects within the IPA knowledge base. Mapped genes, called focus genes, were overlaid onto a global molecular network using information supplied by the IPA knowledge base. Focus gene networks were algorithmically generated based on connectivity. Biological function was assigned to each network based on specific focus genes identified in the IPA knowledge base. Fisher’s exact test was used to determine the probability that each function assigned to a network was not due to chance alone.

### MAP Functional network and pathway analyses

MAP transcripts identified by RNA-seq mapping that had a *P* < 0.05 were considered for network analyses. MAP gene identification numbers were uploaded into Search Tool for the Retrieval of Interacting Genes (STRING) ver. 9.0 and examined for gene networks [119]. Gene connections were created based on direct (physical) and indirect (functional) interactions derived from genomic context, high-throughput experimentation, coexpression and knowledge reported in literature. MAP networks identified by STRING ver. 9.0 were analyzed for pathways. Pathways were established by extensive domain searches and annotated functions, which were compared to the Kyoto Encyclopedia of Genes and Genomes (KEGG) pathways and knowledge reported in the literature including information published on *M. tuberculosis*.

### Interactome development and functional analysis

As previously described, DGE expression for bovine and MAP transcripts was determined by Cuffdiff and edgeR, respectively. DGE data for co-cultured conditions versus a single cell type during MAP infection (both host and pathogen transcripts) were combined. A cluster analysis was performed using the k-means algorithm to visualize expression patterns shared between bovine and MAP transcripts. Biological processes were assigned to bovine genes that correlated with MAP transcripts using Gene Ontology identified by Database for Annotation, Visualization, and Integrated Discovery (DAVID) v6.7 (http://david.abcc.ncifcrf.gov/).

### Quantitative real-time PCR validation

Quantitative real-time PCR (qT-RT-PCR) was conducted on selected host (3 upregulated, 3 downregulated, and 3 non-differentially expressed) genes (Table 6) using the Quantifast One-Step SYBR Green qT-RT-PCR kit (Qiagen, Valencia, CA). All samples were analyzed on a Roche Lightcycler 480II with corresponding software (Roche NimbleGen Inc., Madison, WI). The following cycling program was used: 50°C for 10 min, 95°C for 5 min, 95°C for 10 s and 60°C for 30 s for 40 cycles. Primers were designed using Primer 3 (http://frodo.wi.mit.edu/primer3/). Fold change was calculated using the ^ΔΔ^CT method and the house-keeping gene, *β-actin*, which was normalized to uninfected macrophages. Fold changes were graphed using GraphPad Prism software (GraphPad Software Inc., La Jolla, CA). Correlation coefficient (r^2^) was calculated using GraphPad software. qT-RT-PCR products were examined on a 2% agarose gel. All samples were conducted in triplicate.

**Table 6:** Primers used for qT-RT-PCR

**Gene and direction**	**Sequence (5′---3′)**
*fstl1, F*	GTGTGTGTGCCTGTGGAAAC
*fstl1, R*	TCTGATTCTTTCCGTCACAGG
*CD46, F*	GGTACCCTTAAACCCAGTTATAGTCC
*CD46, R*	CTGGAAACCCAGACGACATT
*B2M, F*	AAGGATGGCTCGCTTCGT
*B2M, R*	GCGTCCAGTCCAGACAGC
*BCAM, F*	GGATCCCCCTCCTGAGTC
*BCAM, R*	ACTCTGGTGTCCCTTGAACC
*DHRSI3, F*	CCAGACACCCCTGTACTGC
*DHRSI3, R*	CGAAGTACCTCCCGCTGAG
*B-actin, F*	TCCTCCCTGGAGAAGAGCTA
*B-actin, R*	GTAGAGGTCCTTGCGGATGT
*CD274, F*	GCGATCACCAAGTCCTGAGT
*CD274, R*	GCTTTTCCTCCCTCTTTGAAC
*TNFAIP3, F*	AGATGAAGGAAAAGCTCCTGAA
*TNFAIP3, R*	AGCCTTGAACGGGGATTT
*IFITM1, F*	TCTAGGGACCGGAAGATGGT
*IFITM1, R*	ACTTGGCGGTAGAGGCGTA

## Competing interests

The authors claim no competing interests.

## Authors’ contributions

EAL performed experiments. EAL, WWX, and SS analyzed and interpreted data. EAL and SS wrote the paper. SS provided financial support. All authors read and approved the final manuscript.

## Supplementary Material

Additional file 1 **Sheets 1-12: Differentially expressed genes identified by RNA-seq.** Total read counts. S1 is uninfected MAC-T cells alone, S2 is uninfected macrophages alone, S3 is MAP infected MAC-T cells alone, S4 is MAP infected macrophages alone, S5 is co-cultured MAC-T cells, S6 is co-cultured macrophages, S7 is MAP infected co-cultured MAC-T cells, and S8 is MAP infected co-cultured macrophages. Fold changes are calculated based on infection status and culture comparisons. Genes shown have a P < 0.05. Click here for file

Additional file 2 **Downregulation of protein synthesis and cell cycle network in uninfected co-cultured MAC-T cells.** Downregulated genes are shown in green. Upregulated genes are shown in red. Color intensity reflects degree of downregulation/upregulation. Solid lines represent direct relationships. Dotted lines represent indirect relationships. Genes shown have a P < 0.05. Click here for file

Additional file 3 **Downregulation of assembly and organization, cellular function and maintenance, nucleic acid metabolism network in uninfected co-cultured MAC-T cells compared to uninfected MAC-T cells cultured alone.** Downregulated genes are shown in green. Upregulated genes are shown in red. Color intensity reflects degree of downregulation/upregulation. Solid lines represent direct relationships. Dotted lines represent indirect relationships. Genes shown have a P < 0.05. Click here for file

Additional file 4 **Downregulation of network involved in cancer, dermatological diseases and conditions and lymphoid tissue structure and development in uninfected co-cultured MAC-T cells compared to uninfected MAC-T cells cultured alone.** Downregulated genes are shown in green. Color intensity reflects degree of downregulation. Solid lines represent direct relationships. Dotted lines represent indirect relationships. Genes shown have a P < 0.05. Click here for file

Additional file 5 **Downregulation of cell cycle, cell morphology, and cellular assembly and organization network in uninfected co-cultured MAC-T cells compared to uninfected MAC-T cells cultured alone.** Downregulated genes are shown in green. Color intensity reflects degree of downregulation. Solid lines represent direct relationships. Dotted lines represent indirect relationships. Genes shown have a P < 0.05. Click here for file

Additional file 6: **Downregulation of inflammatory disease network in uninfected co-cultured macrophages compared to uninfected macrophages cultured alone.** The majority of differentially expressed genes identified in the inflammatory disease network were downregulated. Downregulated genes are shown in green. Upregulated genes are shown in red. Color intensity reflects degree of downregulation/upregulation. Solid lines represent direct relationships. Dotted lines represent indirect relationships. Genes shown have a P < 0.05. Click here for file

Additional file 7 **Downregulation of molecular transport network in uninfected co-cultured macrophages compared to uninfected macrophages cultured alone.** The majority of differentially expressed genes identified in the molecular transport network were downregulated. Downregulated genes are shown in green. Upregulated genes are shown in red. Color intensity reflects degree of downregulation/upregulation. Solid lines represent direct relationships. Dotted lines represent indirect relationships. Genes shown have a P < 0.05. Click here for file

Additional file 8 **Downregulation of infectious disease network in uninfected co-cultured macrophages compared to uninfected macrophages cultured alone.** The majority of differentially expressed genes identified in the infectious disease network were downregulated. Downregulated genes are shown in green. Upregulated genes are shown in red. Color intensity reflects degree of downregulation/upregulation. Solid lines represent direct relationships. Dotted lines represent indirect relationships. Genes shown have a P < 0.05. Click here for file

Additional file 9 **Upregulation of cellular growth and proliferation network in response to MAP infection in co-cultured MAC-T cells compared to infection in MAC-T cells alone.** The majority of differentially expressed genes were upregulated. Downregulated genes are shown in green. Upregulated genes are shown in red. Color intensity reflects degree of downregulation/upregulation. Solid lines represent direct relationships. Dotted lines represent indirect relationships. Genes shown have a P < 0.05. Click here for file

Additional file 10 **Upregulation of DNA replication, recombination and repair in response to MAP infection in co-cultured MAC-T cells compared to infection in MAC-T cells alone.** All differentially expressed genes found in this network were upregulated. Upregulated genes are shown in red. Color intensity reflects degree of downregulation/upregulation. Solid lines represent direct relationships. Dotted lines represent indirect relationships. Genes shown have a P < 0.05. Click here for file

Additional file 11 **Downregulation of cell death network in response to MAP infection in co-cultured macrophages vs. infected macrophages alone.** Downregulated genes are shown in green. Upregulated genes are shown in red. Color intensity reflects degree of downregulation/upregulation. Solid lines represent direct relationships. Dotted lines represent indirect relationships. Genes shown have a P < 0.05. Click here for file

Additional file 12 **Upregulation of cell to cell communication and signaling in response to Map infection in co-cultured macrophages vs. infected macrophages alone.** Downregulated genes are shown in green. Upregulated genes are shown in red. Color intensity reflects degree of downregulation/upregulation. Solid lines represent direct relationships. Dotted lines represent indirect relationships. Genes shown have a P < 0.05. Click here for file

Additional file 13 **Network Analysis of differentially expressed MAP genes during MAC-T cells infection vs. macrophage infection.** Genes shown have a P < 0.05. STRING software depicts the following relationships by colored lines: neighborhood = green, gene fusion = red, co-occurrence = blue, experiments = pink, databases = turquoise, textmining = yellow and homology = periwinkle. Click here for file

Additional file 14 **Network Analysis of differentially expressed MAP genes during MAC-T cell infection cultured alone vs. MAC-T cells under co-cultured conditions.** Genes shown have a P < 0.05. STRING software depicts the following relationships by colored lines: neighborhood = green, gene fusion = red, co-occurrence = blue, experiments = pink, databases = turquoise, textmining = yellow and homology = periwinkle. Click here for file

Additional file 15 **Network Analysis of differentially expressed MAP genes in co-cultured MAC-T cells vs. co-cultured macrophages.** Genes shown have a P < 0.05. STRING software depicts the following relationships by colored lines: neighborhood = green, gene fusion = red, co-occurrence = blue, experiments = pink, databases = turquoise, textmining = yellow and homology = periwinkle. Click here for file

Additional file 16 **K-clustering analysis.** K-clustering analysis identified 81 host genes that shared a similar regulation pattern profile with 77 MAP genes. b3 = MAP genes in MAC-T cells alone, b7 = MAP genes in co-cultured MAC-T cells, b4 = MAP genes in macrophages alone, b8 = MAP genes in co-cultured macrophages, m3 = MAP genes in MAC-T cells alone, m7 = MAP genes in co-cultured MAC-T cells, m4 = MAP genes in macrophages alone, and m8 = MAP genes in co-cultured macrophages. Click here for file

Additional file 17 **Interactome network analysis.** Genes shown have a P < 0.05. STRING software depicts the following relationships by colored lines: neighborhood = green, gene fusion = red, co-occurrence = blue, experiments = pink, databases = turquoise, textmining = yellow and homology = periwinkle. Click here for file

Additional file 18 **RNA-seq quality control.** Bovine and MAP profiles were analyzed in Galaxy using the Tuxedo Suite Tools. A) Average and individual reads for bovine transcripts had a Phred score above 30. B) Principal component analysis (PCA) of bovine transcripts. All transcripts stratified according to cell type. C) Average and individual reads for MAP transcripts had a Phred score above 30. D) PCA shows all MAP transcripts stratified according to infected cell type. Click here for file
