# Supplementary figures and images for: Host-Mycobacterium avium subsp. paratuberculosis interactome reveals a novel iron assimilation mechanism linked to nitric oxide stress during early infection
Source: BMC Genomics. 2013 Oct 10;14:694. doi: 10.1186/1471-2164-14-694 (PMC3832399; doi:10.1186/1471-2164-14-694)

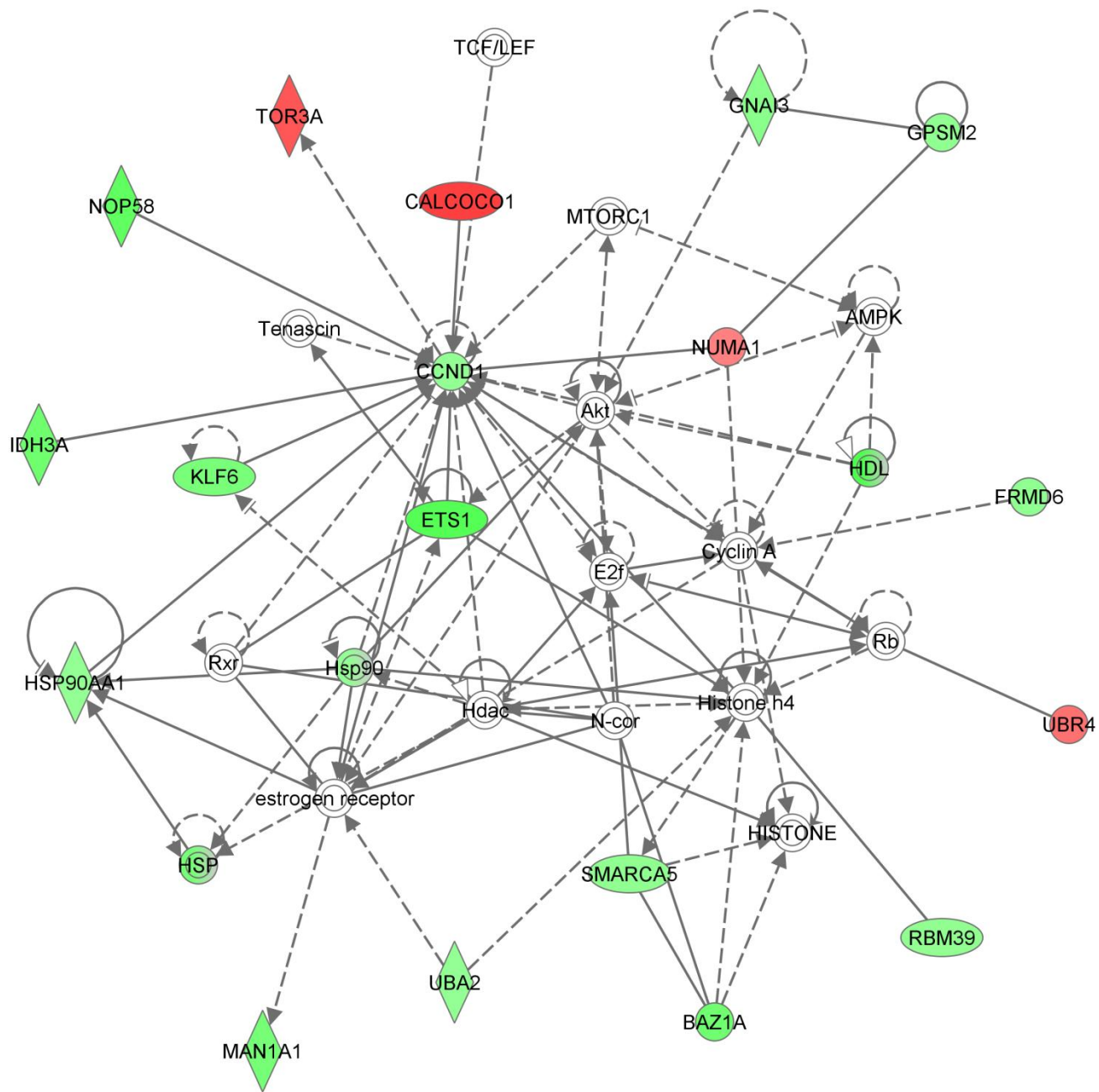

Supplement: Additional file 3 — Downregulation of assembly and organization, cellular function and maintenance, nucleic acid metabolism network in uninfected co-cultured MAC-T cells compared to uninfected MAC-T cells cultured alone. Downregulated genes are shown in green. Upregulated genes are shown in red. Color intensity reflects degree of downregulation/upregulation. Solid lines represent direct relationships. Dotted lines represent indirect relationships. Genes shown have a P < 0.05. [file 1471-2164-14-694-S3.pdf]

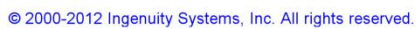

Supplement: Additional file 4 — Downregulation of network involved in cancer, dermatological diseases and conditions and lymphoid tissue structure and development in uninfected co-cultured MAC-T cells compared to uninfected MAC-T cells cultured alone. Downregulated genes are shown in green. Color intensity reflects degree of downregulation. Solid lines represent direct relationships. Dotted lines represent indirect relationships. Genes shown have a P < 0.05. [file 1471-2164-14-694-S4.pdf]

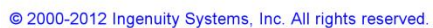

Supplement: Additional file 6: — Downregulation of inflammatory disease network in uninfected co-cultured macrophages compared to uninfected macrophages cultured alone. The majority of differentially expressed genes identified in the inflammatory disease network were downregulated. Downregulated genes are shown in green. Upregulated genes are shown in red. Color intensity reflects degree of downregulation/upregulation. Solid lines represent direct relationships. Dotted lines represent indirect relationships. Genes shown have a P < 0.05. [file 1471-2164-14-694-S6.pdf]

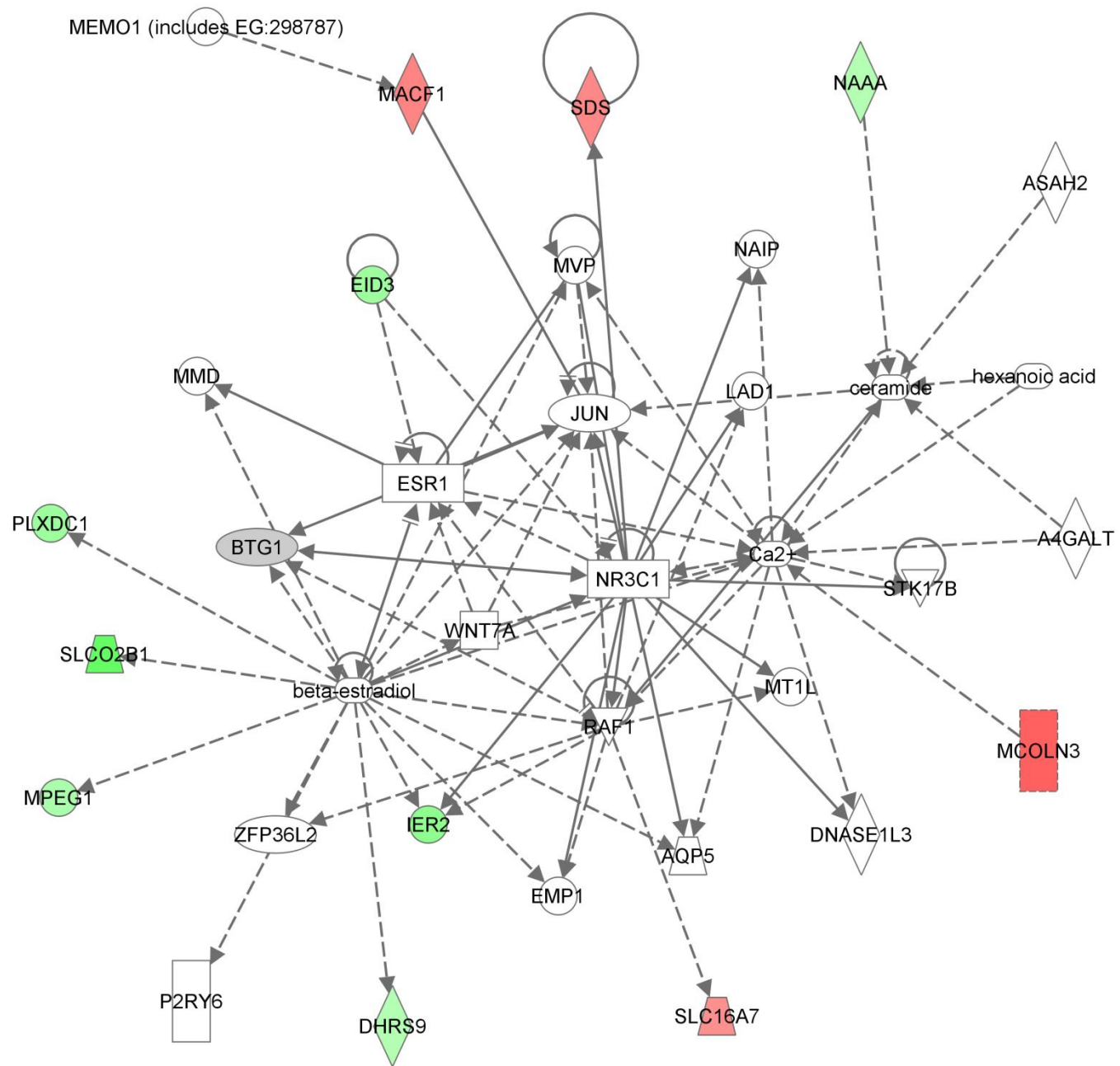

Supplement: Additional file 7 — Downregulation of molecular transport network in uninfected co-cultured macrophages compared to uninfected macrophages cultured alone. The majority of differentially expressed genes identified in the molecular transport network were downregulated. Downregulated genes are shown in green. Upregulated genes are shown in red. Color intensity reflects degree of downregulation/upregulation. Solid lines represent direct relationships. Dotted lines represent indirect relationships. Genes shown have a P < 0.05. [file 1471-2164-14-694-S7.pdf]

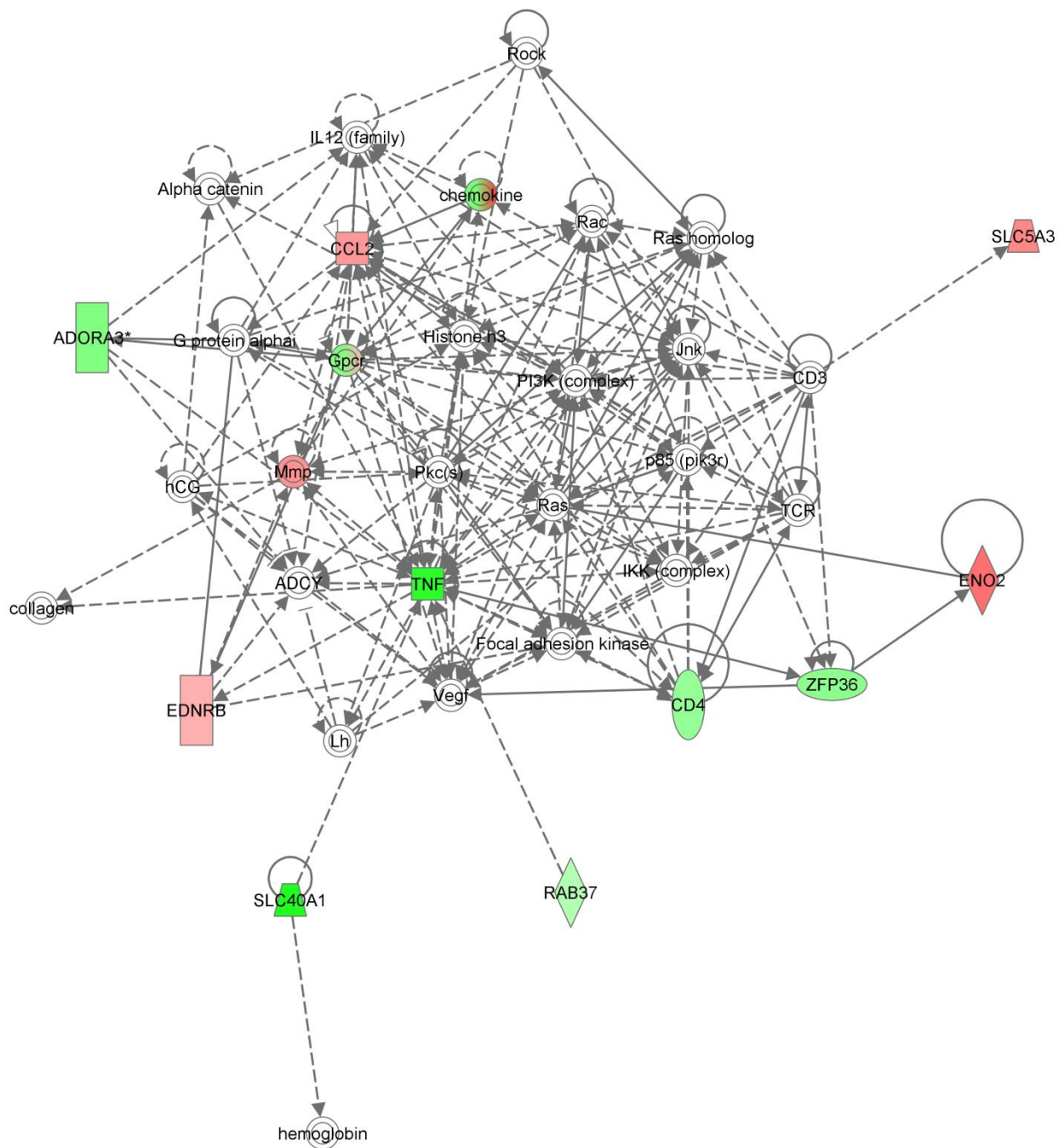

Supplement: Additional file 8 — Downregulation of infectious disease network in uninfected co-cultured macrophages compared to uninfected macrophages cultured alone. The majority of differentially expressed genes identified in the infectious disease network were downregulated. Downregulated genes are shown in green. Upregulated genes are shown in red. Color intensity reflects degree of downregulation/upregulation. Solid lines represent direct relationships. Dotted lines represent indirect relationships. Genes shown have a P < 0.05. [file 1471-2164-14-694-S8.pdf]

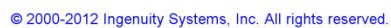

Supplement: Additional file 9 — Upregulation of cellular growth and proliferation network in response to MAP infection in co-cultured MAC-T cells compared to infection in MAC-T cells alone. The majority of differentially expressed genes were upregulated. Downregulated genes are shown in green. Upregulated genes are shown in red. Color intensity reflects degree of downregulation/upregulation. Solid lines represent direct relationships. Dotted lines represent indirect relationships. Genes shown have a P < 0.05. [file 1471-2164-14-694-S9.pdf]

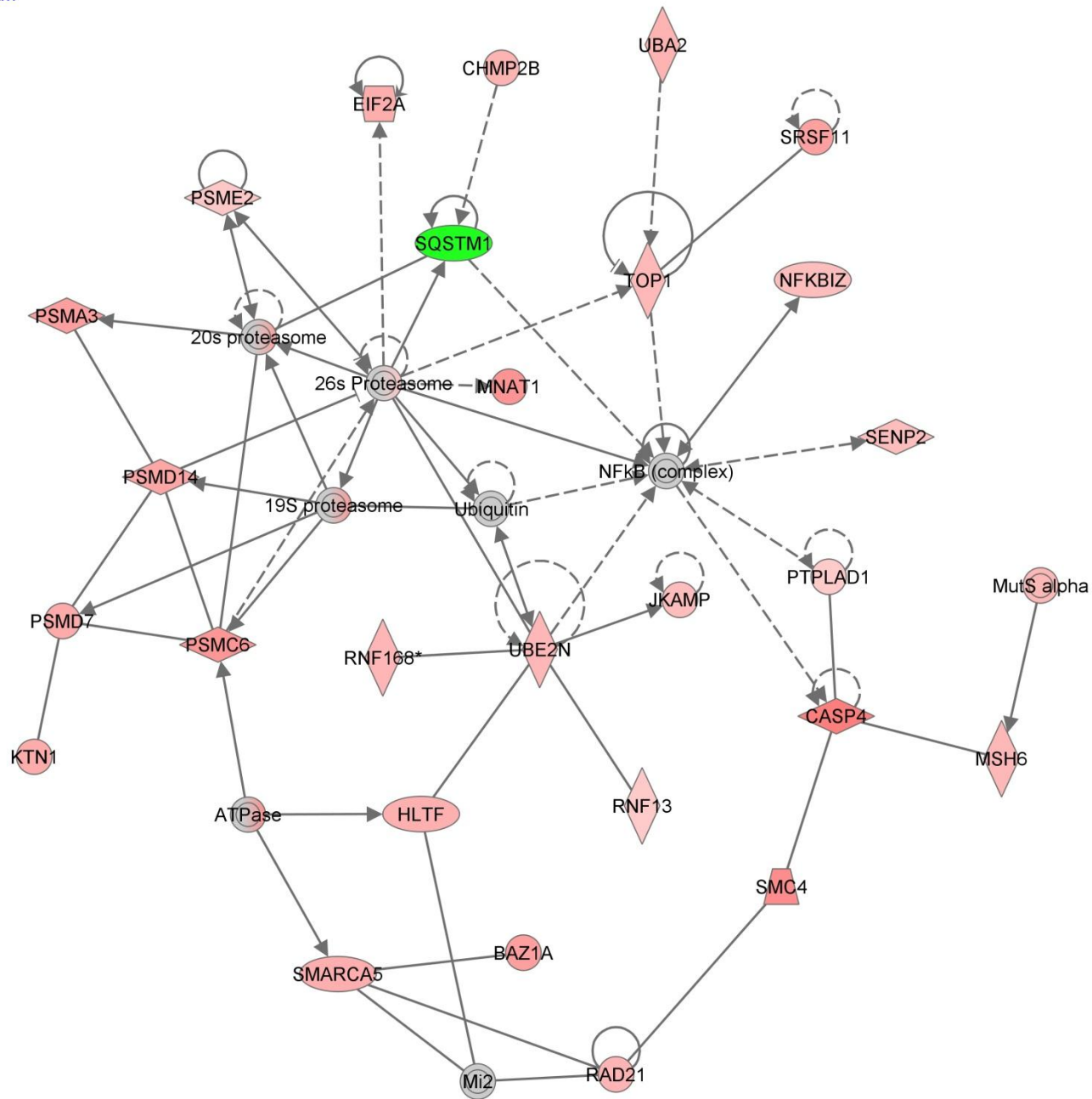

Supplement: Additional file 10 — Upregulation of DNA replication, recombination and repair in response to MAP infection in co-cultured MAC-T cells compared to infection in MAC-T cells alone. All differentially expressed genes found in this network were upregulated. Upregulated genes are shown in red. Color intensity reflects degree of downregulation/upregulation. Solid lines represent direct relationships. Dotted lines represent indirect relationships. Genes shown have a P < 0.05. [file 1471-2164-14-694-S10.pdf]

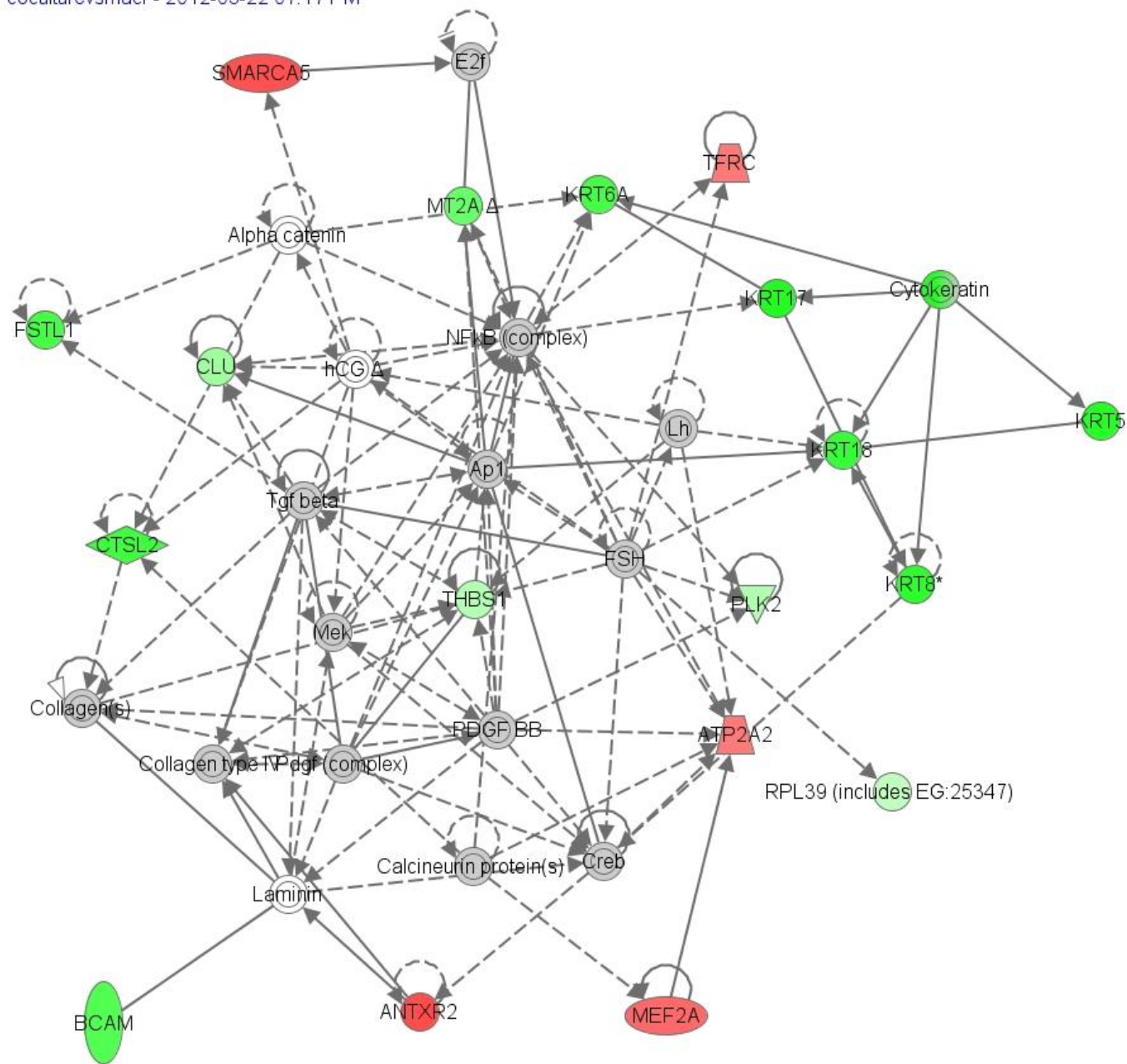

Supplement: Additional file 11 — Downregulation of cell death network in response to MAP infection in co-cultured macrophages vs. infected macrophages alone. Downregulated genes are shown in green. Upregulated genes are shown in red. Color intensity reflects degree of downregulation/upregulation. Solid lines represent direct relationships. Dotted lines represent indirect relationships. Genes shown have a P < 0.05. [file 1471-2164-14-694-S11.pdf]

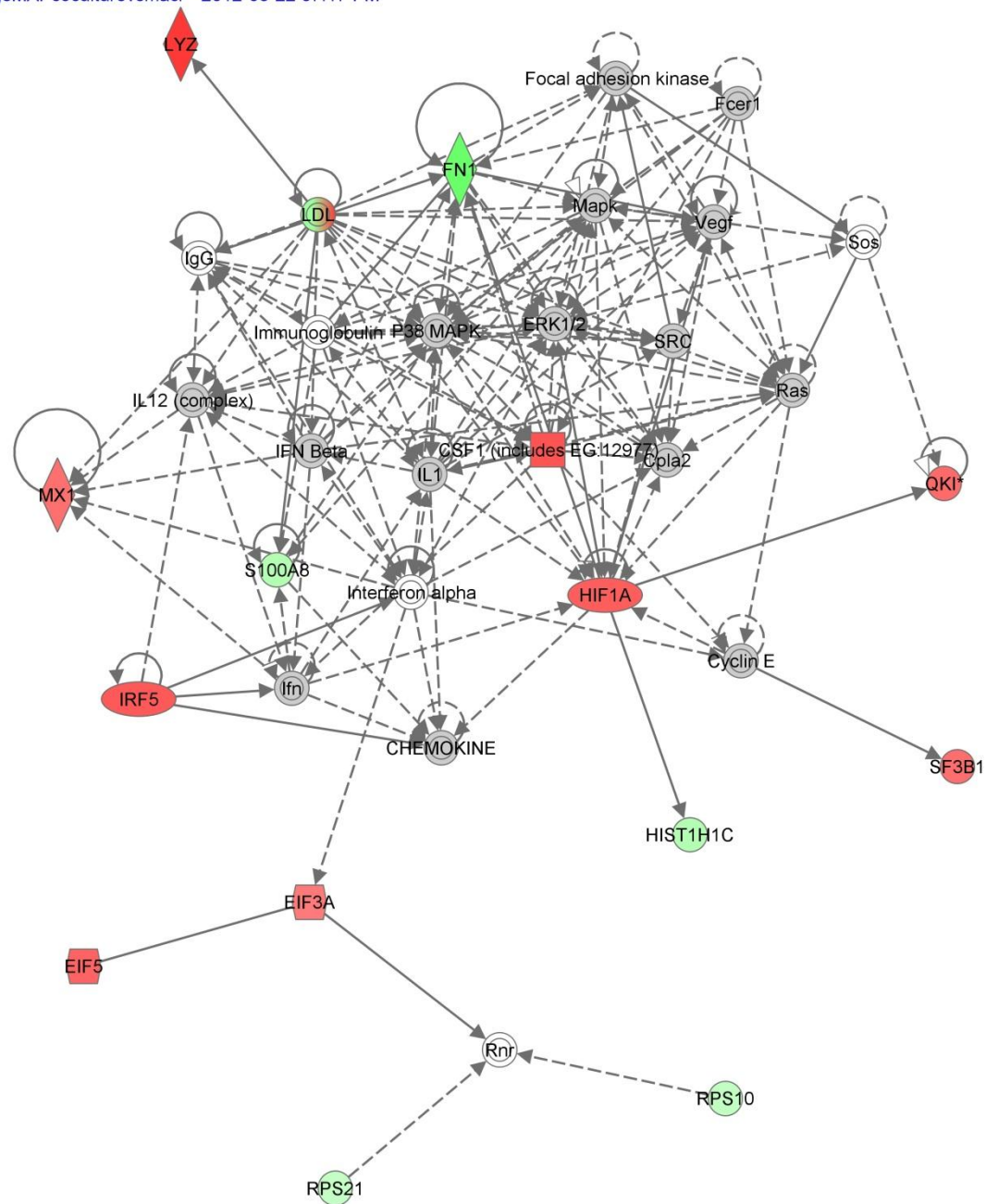

Supplement: Additional file 12 — Upregulation of cell to cell communication and signaling in response to Map infection in co-cultured macrophages vs. infected macrophages alone. Downregulated genes are shown in green. Upregulated genes are shown in red. Color intensity reflects degree of downregulation/upregulation. Solid lines represent direct relationships. Dotted lines represent indirect relationships. Genes shown have a P < 0.05. [file 1471-2164-14-694-S12.pdf]

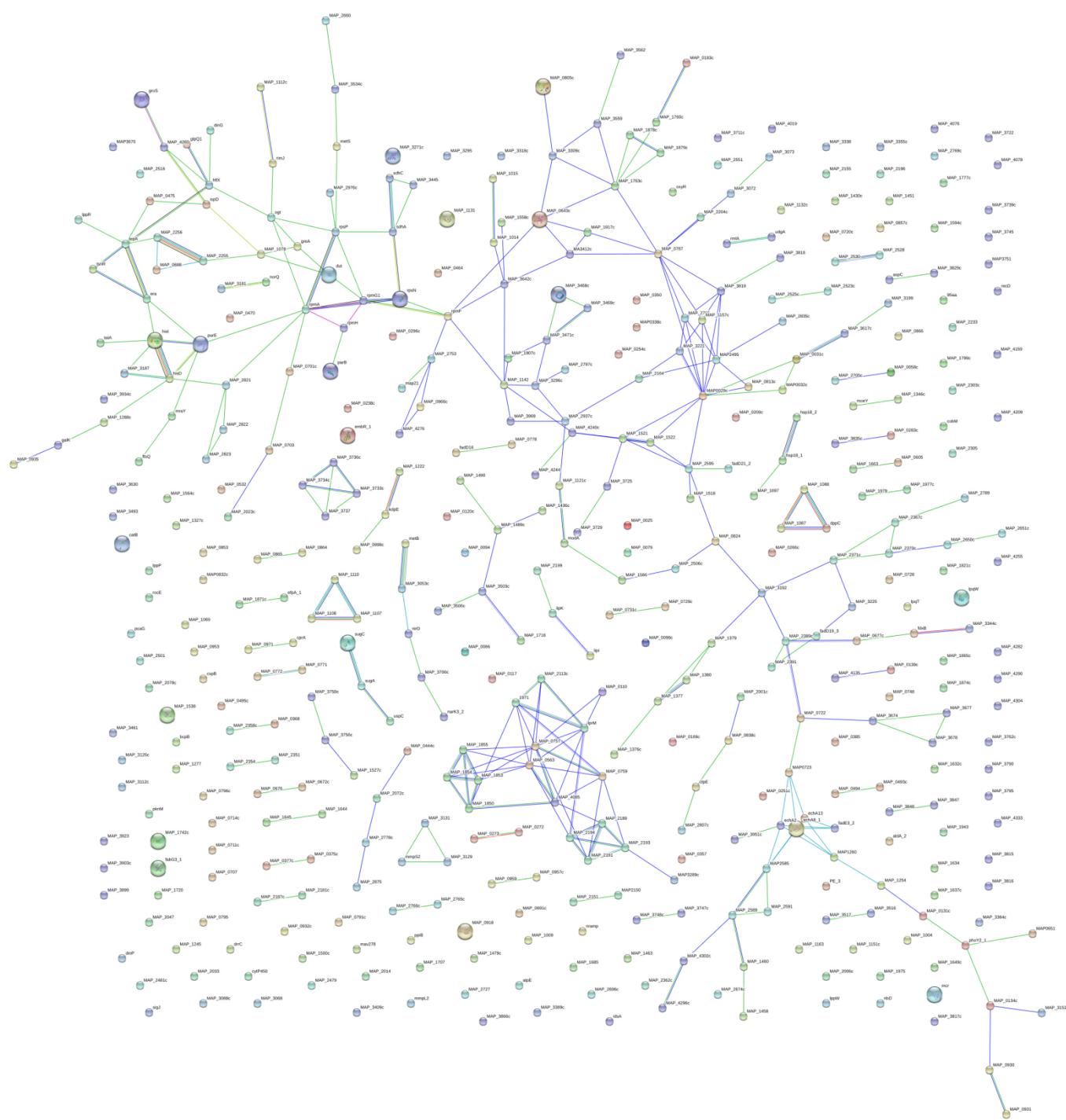

Supplement: Additional file 13 — Network Analysis of differentially expressed MAP genes during MAC-T cells infection vs. macrophage infection. Genes shown have a P < 0.05. STRING software depicts the following relationships by colored lines: neighborhood = green, gene fusion = red, co-occurrence = blue, experiments = pink, databases = turquoise, textmining = yellow and homology = periwinkle. [file 1471-2164-14-694-S13.pdf]

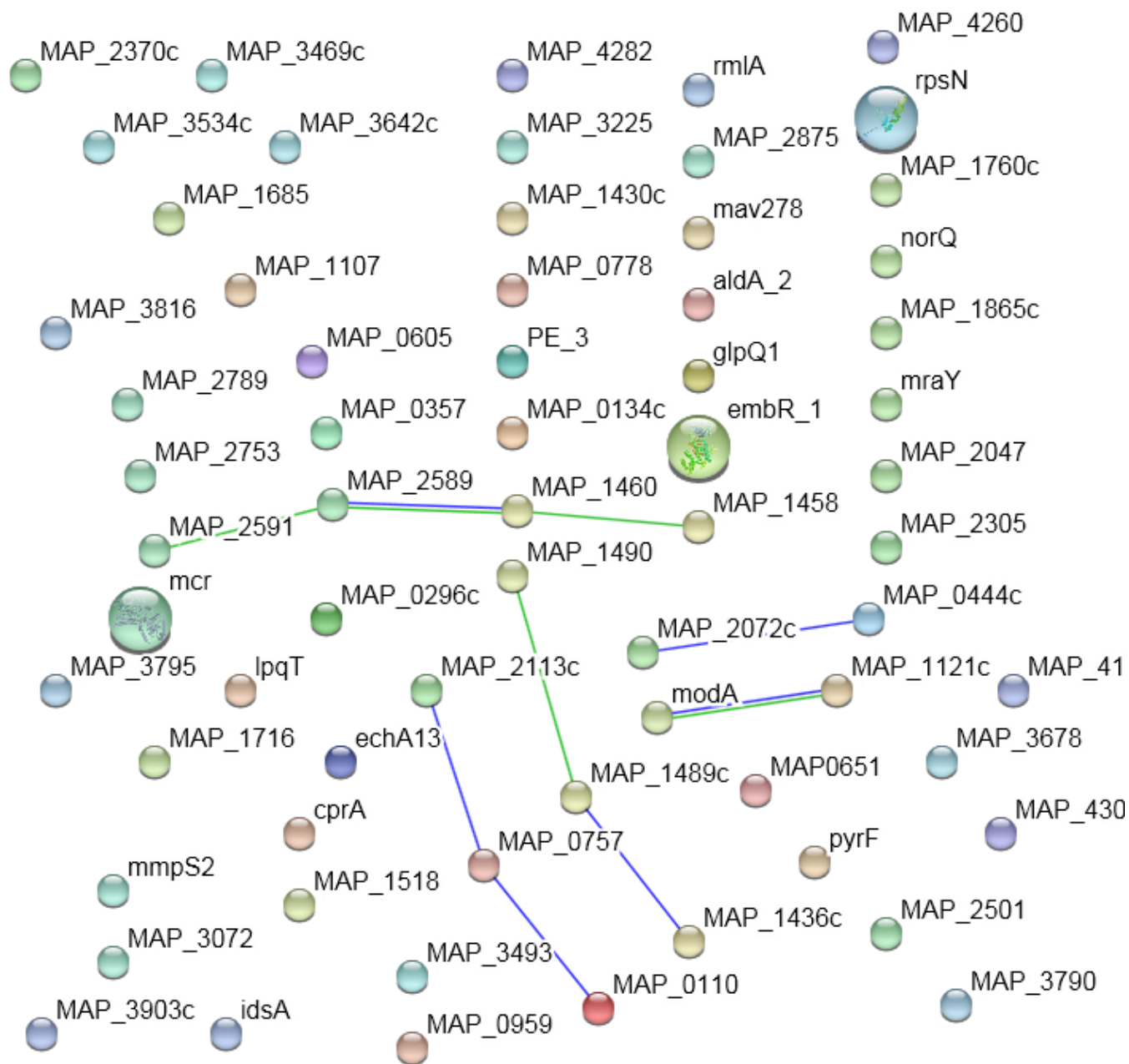

Supplement: Additional file 14 — Network Analysis of differentially expressed MAP genes during MAC-T cell infection cultured alone vs. MAC-T cells under co-cultured conditions. Genes shown have a P < 0.05. STRING software depicts the following relationships by colored lines: neighborhood = green, gene fusion = red, co-occurrence = blue, experiments = pink, databases = turquoise, textmining = yellow and homology = periwinkle. [file 1471-2164-14-694-S14.pdf]

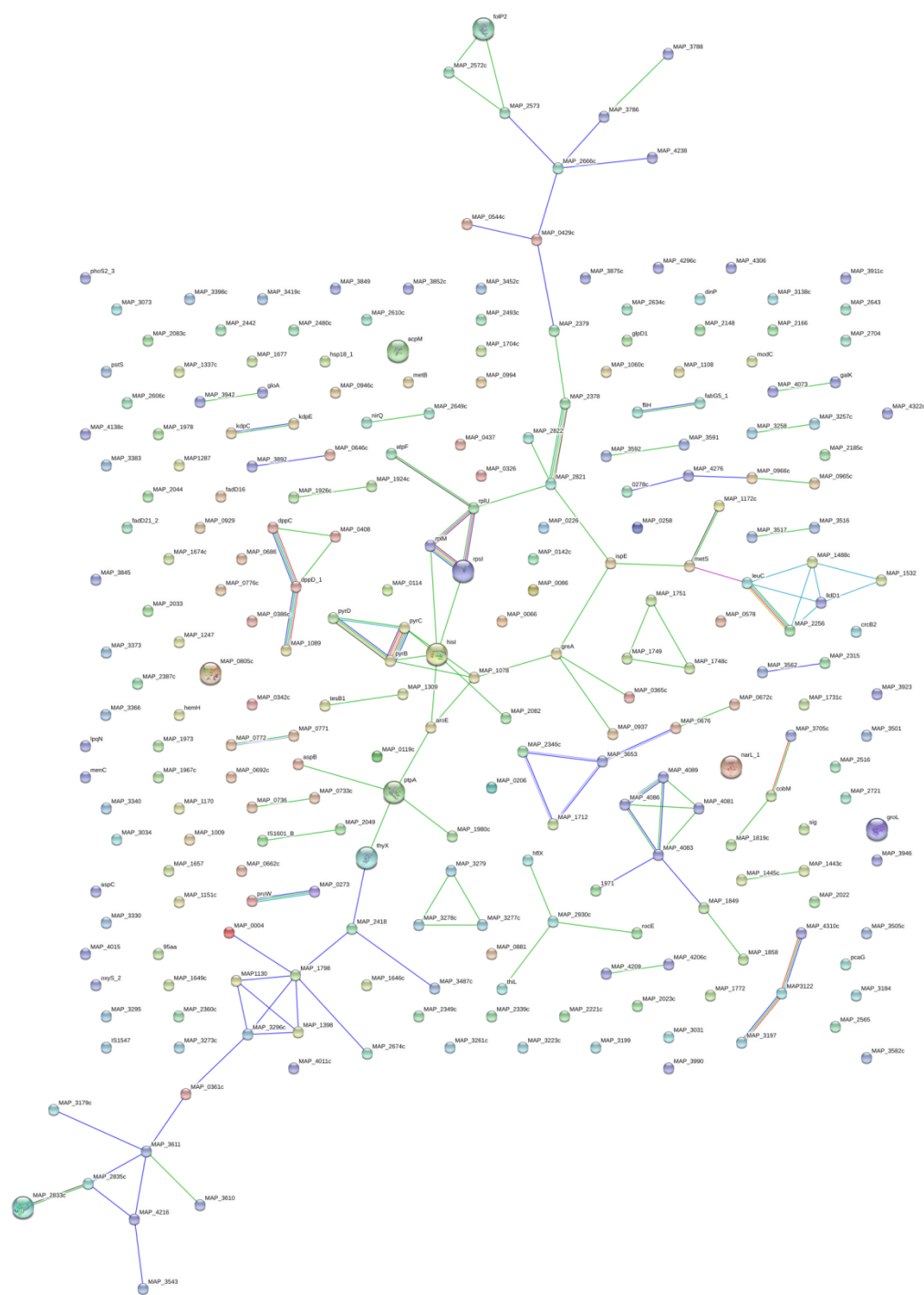

Supplement: Additional file 15 — Network Analysis of differentially expressed MAP genes in co-cultured MAC-T cells vs. co-cultured macrophages. Genes shown have a P < 0.05. STRING software depicts the following relationships by colored lines: neighborhood = green, gene fusion = red, co-occurrence = blue, experiments = pink, databases = turquoise, textmining = yellow and homology = periwinkle. [file 1471-2164-14-694-S15.pdf]

**Column**

B3  
B7  
m3  
m7  
B4  
B8  
m4  
m8

**Row**

From 66: K-Means

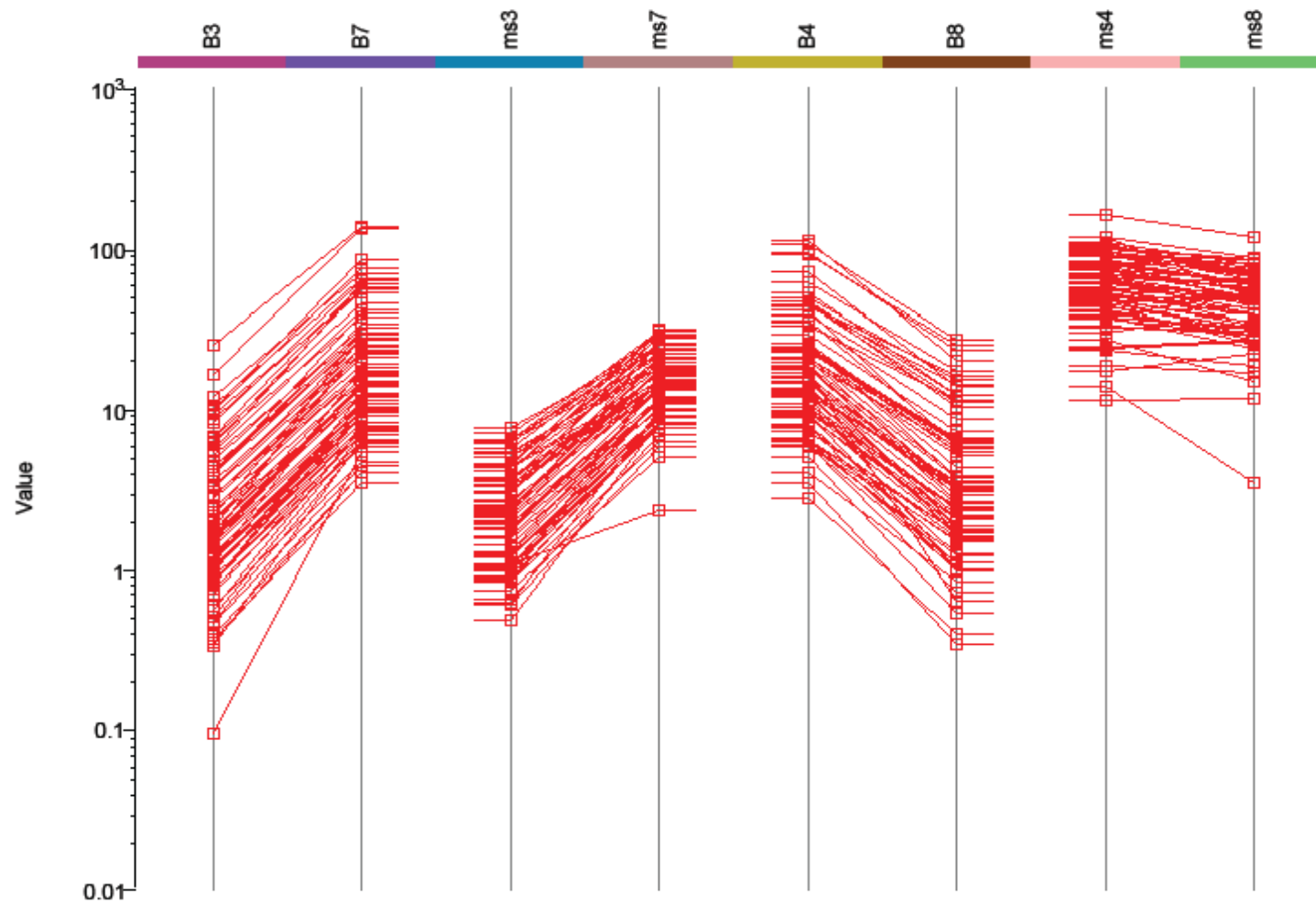

Supplement: Additional file 16 — K-clustering analysis. K-clustering analysis identified 81 host genes that shared a similar regulation pattern profile with 77 MAP genes. b3 = MAP genes in MAC-T cells alone, b7 = MAP genes in co-cultured MAC-T cells, b4 = MAP genes in macrophages alone, b8 = MAP genes in co-cultured macrophages, m3 = MAP genes in MAC-T cells alone, m7 = MAP genes in co-cultured MAC-T cells, m4 = MAP genes in macrophages alone, and m8 = MAP genes in co-cultured macrophages. [file 1471-2164-14-694-S16.pdf]

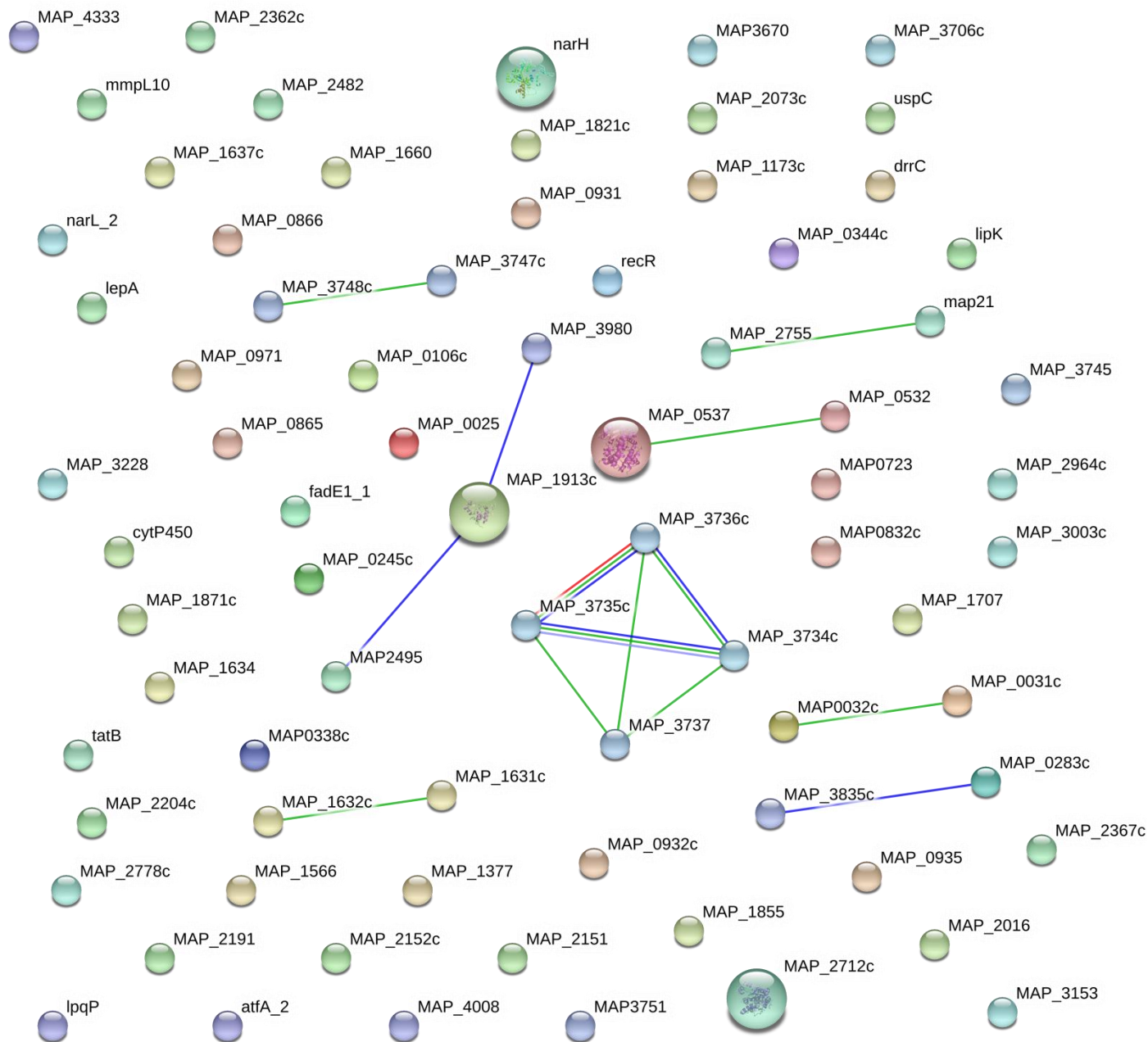

Supplement: Additional file 17 — Interactome network analysis. Genes shown have a P < 0.05. STRING software depicts the following relationships by colored lines: neighborhood = green, gene fusion = red, co-occurrence = blue, experiments = pink, databases = turquoise, textmining = yellow and homology = periwinkle. [file 1471-2164-14-694-S17.pdf]

A)

Host-Bovine

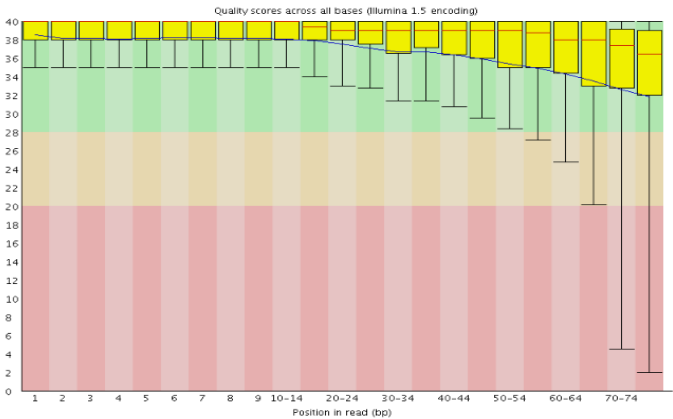

Pathogen-MAP

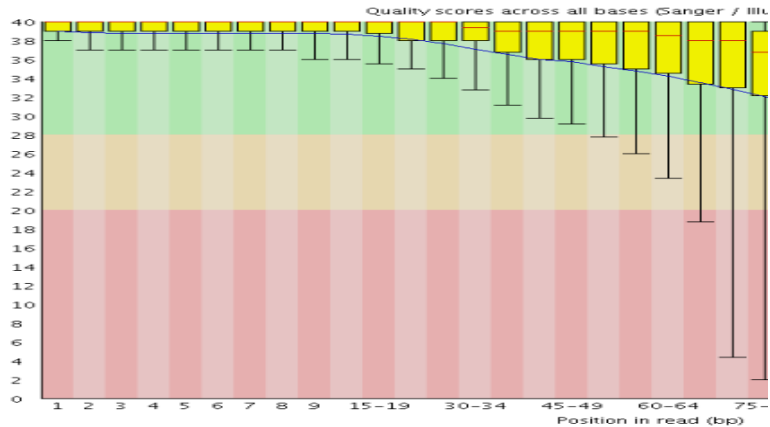

B)

Host-Bovine

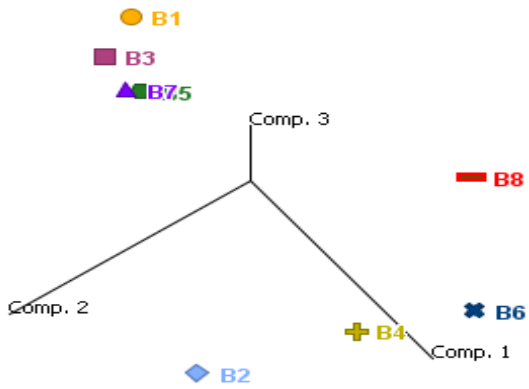

Pathogen-MAP

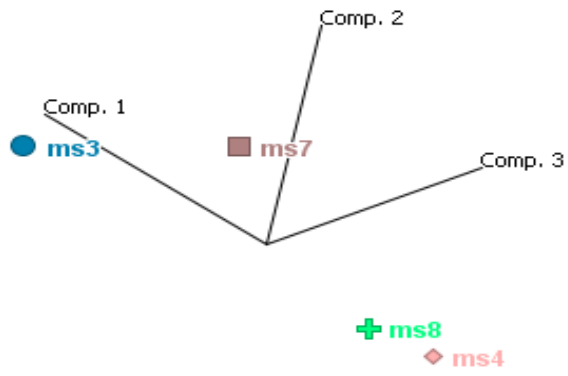

Supplement: Additional file 18 — RNA-seq quality control. Bovine and MAP profiles were analyzed in Galaxy using the Tuxedo Suite Tools. A) Average and individual reads for bovine transcripts had a Phred score above 30. B) Principal component analysis (PCA) of bovine transcripts. All transcripts stratified according to cell type. C) Average and individual reads for MAP transcripts had a Phred score above 30. D) PCA shows all MAP transcripts stratified according to infected cell type. [file 1471-2164-14-694-S18.pdf]
