# Supplementary material for: Host-Mycobacterium avium subsp. paratuberculosis interactome reveals a novel iron assimilation mechanism linked to nitric oxide stress during early infection
Source: BMC Genomics. 2013 Oct 10;14:694. doi: 10.1186/1471-2164-14-694 (PMC3832399; doi:10.1186/1471-2164-14-694)
Supplement: Additional file 2 — Downregulation of protein synthesis and cell cycle network in uninfected co-cultured MAC-T cells. Downregulated genes are shown in green. Upregulated genes are shown in red. Color intensity reflects degree of downregulation/upregulation. Solid lines represent direct relationships. Dotted lines represent indirect relationships. Genes shown have a P < 0.05. [file 1471-2164-14-694-S2.pdf]

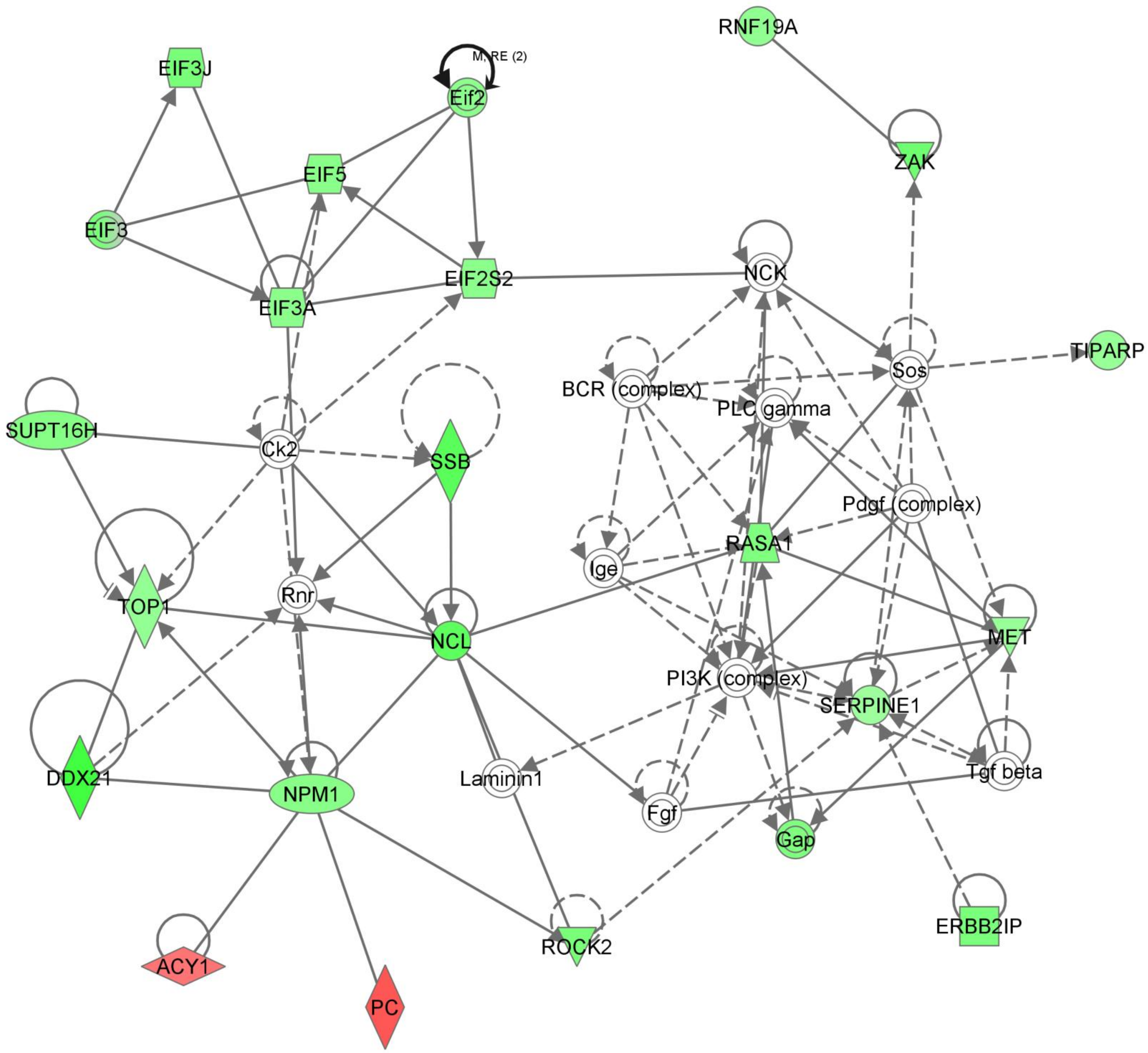

© 2000-2012 Ingenuity Systems, Inc. All rights reserved.
