# Supplementary material for: Host-Mycobacterium avium subsp. paratuberculosis interactome reveals a novel iron assimilation mechanism linked to nitric oxide stress during early infection
Source: BMC Genomics. 2013 Oct 10;14:694. doi: 10.1186/1471-2164-14-694 (PMC3832399; doi:10.1186/1471-2164-14-694)
Supplement: Additional file 5 — Downregulation of cell cycle, cell morphology, and cellular assembly and organization network in uninfected co-cultured MAC-T cells compared to uninfected MAC-T cells cultured alone. Downregulated genes are shown in green. Color intensity reflects degree of downregulation. Solid lines represent direct relationships. Dotted lines represent indirect relationships. Genes shown have a P < 0.05. [file 1471-2164-14-694-S5.pdf]

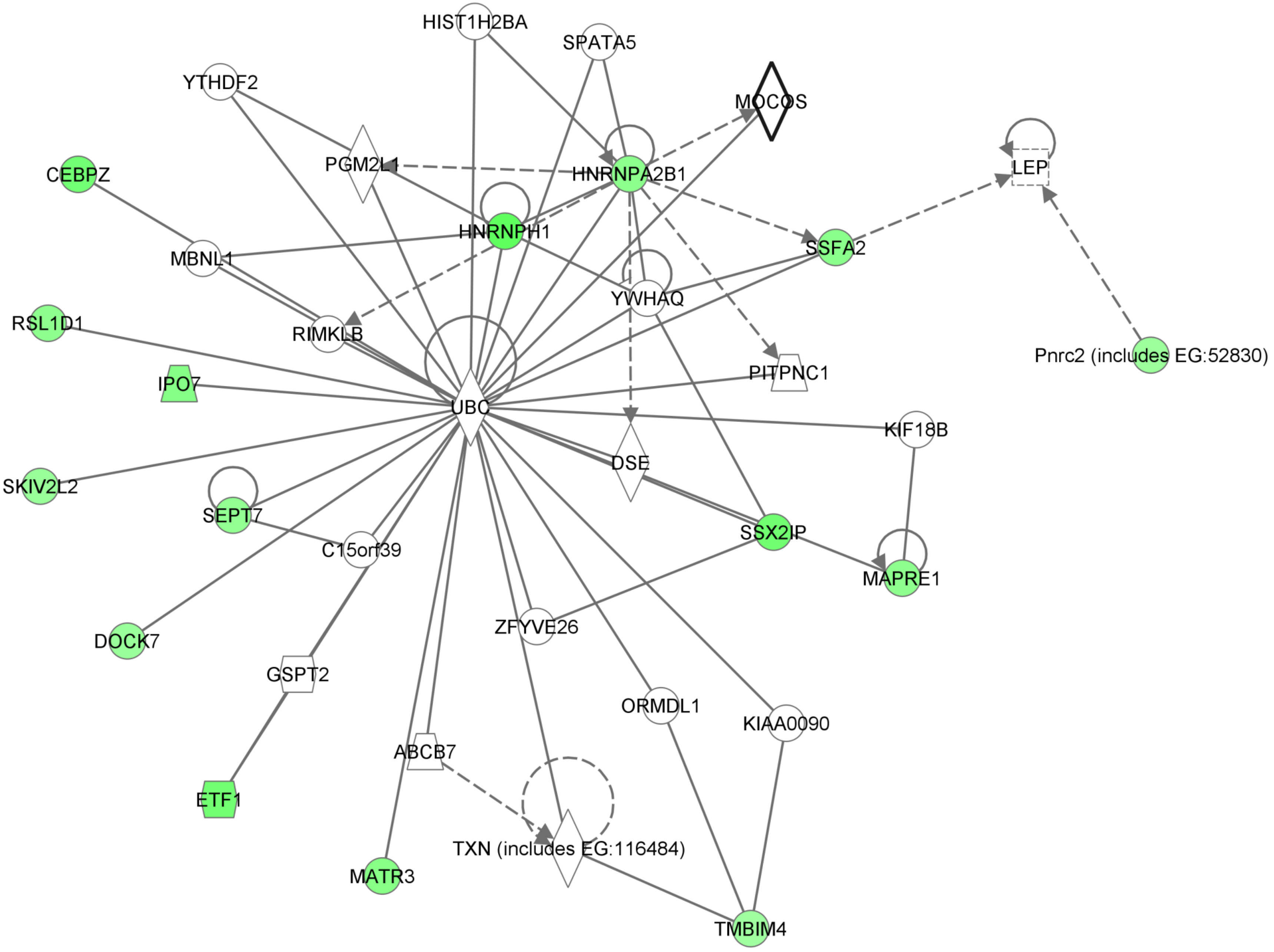

© 2000-2012 Ingenuity Systems, Inc. All rights reserved.
